# *Megasphaera Elesdenii* Dysregulates Colon Epithelial Homeostasis, Aggravates Colitis‐Associated Tumorigenesis

**DOI:** 10.1002/advs.202505670

**Published:** 2025-08-13

**Authors:** Xinxin Hou, Zhaozhou Zhang, Wanqing Chen, Jinming Li, Xiangxiang Zhu, Mingjie Li, Xiaoqi Guan, Haidong Guo, Yanlei Ma, Ling Zhao

**Affiliations:** ^1^ School of Integrative Medicine Shanghai University of Traditional Chinese Medicine Shanghai 201203 China; ^2^ Department of Oncology Shanghai Municipal Hospital of Traditional Chinese Medicine Shanghai 200071 China; ^3^ Department of Colorectal Surgery Fudan University Shanghai Cancer Center Shanghai 200032 China; ^4^ Department of Oncology, Shanghai Medical College Fudan University Shanghai 200032 China

**Keywords:** colitis‐associated tumorigenesis, colonic inflammation, dendritic cells, Megasphaera

## Abstract

Altered gut microbiota has evidenced to be associated with colitis and colonic tumorigenesis, however bacteria‐driven mechanism underlying inflammation‐related colon pathologies remains undetermined. This study identifies a previously overlooked genus, *Megasphaera*, is highly abundant in inflammatory bowel diseases (IBD) and colorectal cancer (CRC) cohorts. A *Megasphaera* species, *M. elsdenii* reshapes colonic immunity by triggering dendritic cell (DC)‐mediated Th1 and Th17 inflammation. Mechanistically, *M. elsdenii* and its lipopolysaccharide triggers DC activation via TLR4/NF‐κB/IRF4 pathway. Accordingly, in *Tlr4^−/−^* mice, *M. elsdenii* colonization cannot expand colonic infiltration of DCs and fails to induce Th1 and Th17 responses in. In a Shanghai CRC cohort with a 100% detective rate for *Megasphaera*, the species of *Megasphaera* significantly dominated in early onset CRC patients (<50 years old) rather than late‐onset CRC (≥50 years old). *M. elsdenii* and *M. elsdenii*‐enriched microbiota exacerbate colonic inflammation and tumorigenesis in azoxymethane (AOM)/dextran sodium sulfate (DSS)‐induced murine colitis‐associated cancer model. Taken together, this study uncovers a pathogenic role of *Megasphaera* in developing colonic tumorigenesis via activating DC‐mediated inflammation.

## Graphical abstract

A fecal microbial meta‐analysis reveals that *Megasphaera* is highly abundant in the Asian population with IBD and CRC. *M. elsdenii* reshapes colonic immunity by triggering DC cell‐mediated Th1 and Th17 inflammation. *M. elsdenii* and *M. elsdenii*‐enriched microbiota exacerbate colonic inflammation and tumorigenesis in AOM/DSS mice. *Megasphaera* species are particularly enriched in early‐onset CRC patients.

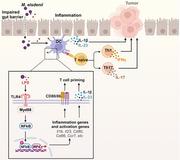

## 1 Introduction

The initiation of colorectal cancer (CRC) is a multistage process resulting from multiple genetic and environmental factors involved in genomic, epigenomic, and transcriptomic alterations.^[^ ^1^, ^2^ ^]^ Colitis‐associated cancer (CAC), a specific cluster of CRCs, develops as a result of prolonged colitis in patients with inflammatory bowel disease (IBD). The disease duration and extent of IBD, as well as the severity of colonic inflammation, increase the risk of developing CAC.^[^ ^3^ ^]^ Long‐term inflammation is a primary driver of colonic tumorigenesis and malignant transformation.^[^ ^4^ ^]^

The pathogenesis of CAC remains incompletely understood, however, activation of an inflammatory process of the colonic mucosa, most likely driven by gut microbiota and defective barrier function, is thought to be a prerequisite for shaping a prone microenvironment to carcinogenesis.^[^ ^5^, ^6^ ^]^ Gut microbiota has been highlighted to interact with host mucosal immunity, while dysregulated gut microbiota can result in aberrant reaction of the host immune system, forming the pathogenetic basis of inflammation and inflammation‐associated cancer.^[^ ^6^, ^7^ ^]^ Pathogens, such as *Fusobacterium nucleatum*, *Escherichia coli* and *Bacteroides fragilis*, have been identified as common causal pathogens in IBD and/or CRC, along with their associated mechanisms in intestinal inflammation and malignancy.^[^ ^8^, ^9^, ^10^ ^]^

Beyond that, certain previously overlooked bacteria from *Veillonellaceae* are shown to be highly abundant in active phase of IBD and CRC.^[^ ^11^, ^12^ ^]^ *Megasphaera*, a genus of family *Veillonellaceae*, is a gram‐negative anaerobic cocci type that has been linked with colon inflammation‐related diseases, such as obesity, prediabetes, rheumatoid arthritis, IBD and CRC, etc.^[^ ^13^, ^14^, ^15^, ^16^, ^17^ ^]^ A species *Megasphaera elsdenii* (*M. elsdenii*) is considered as an opportunistic pathogen associated with bacterial vaginosis,^[^ ^18^ ^]^ immunodeficiency virus infection,^[^ ^19^ ^]^ systematic inflammation^[^ ^20^, ^21^, ^22^ ^]^ and neoplasms.^[^ ^23^ ^]^ Yet, the causal role of *Megasphaera* species in mucosal immunity, inflammation and its associated cancer remains unclear.

To fill the gap, we made a meta‐analysis of human microbiota data with a particular interest in *Megasphaera* across published cohorts. We examined the effect and action mechanism of *M. elsdenii* on the colon morphology and immune landscape. Further, we colonized *M. elsdenii* or *M. elsdenii*‐enriched human microbiota into a chemically induced colon carcinogenesis model to clarify the role of *M. elsdenii* in developing CAC. This study reveals a new mechanistic role of *Megasphaera* spp. in regulating mucosal immunity and colon inflammation‐associated cancer.

## 2 Results

### 2.1 *M. elsdenii* Dysregulates Colon Epithelial Homeostasis

**Figure 1:**
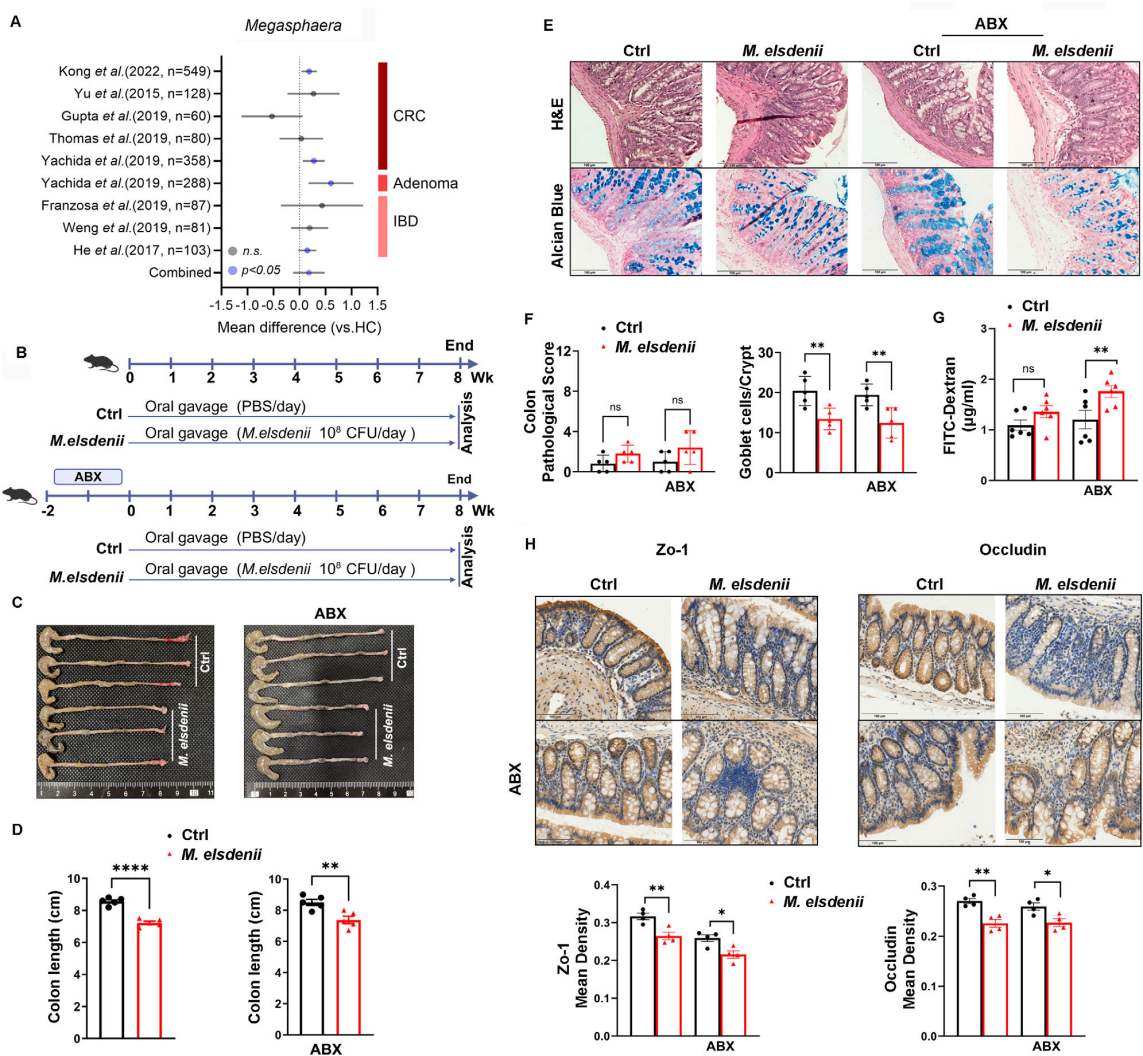
*M. elsdenii* impairs the homeostasis of colon epithelium. A) Random forest (RF) models reporting the effect sizes of genus *Megasphaera* among colorectal cancer (CRC), adenoma, inflammatory bowel disease (IBD) versus healthy control (HC) in indicated cohorts. Horizontal lines represent the 95% confidence interval (CI) for the random effects model estimate. B) Schematic of *Megasphaera elsdenii* (*M. elsdenii*)‐colonized mice protocol. C) Representative macroscopic view of the colons of *M. elsdenii*‐treated in both specific pathogen‐free (SPF) and antibiotic cocktail‐gavaged (ABX) mice. D) Colon length of each group (n = 5/group). E) Representative images of H&E (top) and Alcian blue staining (bottom) of colon tissue paraffin‐embedded sections (n = 5/group), scale bar = 100 µm. F) Pathological score of *M. elsdenii*‐treated colon tissue sections (left) and colon goblet cell count (right) (n = 5/group). G) Serum FITC‐dextran concentration showing in vivo measurement of gut permeability in *M. elsdenii*‐treated mice (n = 6/group). H) Immunohistochemistry staining (IHC) of colon sections by Zo‐1 and Occludin antibody (top). Histograms representing mean density of Zo‐1 and Occludin expression (bottom) (n = 4/group), scale bar = 100 µm. Each experiment conducted 2–3 replicates. Data presented as mean ± SEM. Statistical analysis was performed with Student's t test; ns, not significant, *p < 0.05, **p < 0.01, ****p < 0.0001.

It remains unclear how *Megasphaera* distributes in populations with bowel disease worldwide. To this end, we systematically analyzed published fecal metagenomic or 16S rRNA data within cohorts of IBD and CRC with a particular concern on the relative abundance of *Megasphaera*. Interestingly, *Megasphaera* was detectable in seventeen studies, of which the positive rate large than 25% was shown in seven Asian cohorts only (Figure S1A, Supporting Information). Compared with healthy controls (HC), the *Megasphaera* abundance was found to be highly enriched in IBD, adenoma and CRC patients (**Figure** **1**A). It suggests that *Megasphaera* is more abundant in the gut of Asian populations and plays a potential contributing role in colon inflammation and cancer.

To test the effect of *Megasphaera spp*. in colon epithelial morphology and immunity, we separately colonized the species *M. elsdenii* (oral gavage, 10^8^ CFU per day) in SPF mice and antibiotics (ABX)‐induced pseudo‐germ‐free mice for 8 weeks, while mice treated with PBS serve as controls (Figure 1B). Upon confirming successful depletion of commensal bacteria and colonization of *M. elsdenii* in the gut (Figure S1B,C, Supporting Information), we found that *M. elsdenii* had no impact on body weight and spleen index (Figure S1D,E, Supporting Information), while resulting in a shortened colon length with regardless of commensal bacteria removed or not (Figure 1C,D). We also observed mild epithelial inflammatory changes in *M. elsdenii* group, such as mild submucosal edema, increased immune cell infiltration into the lamina propria (LP) and decreased goblet cells (Figure 1E). These results suggest that *M. elsdenii* could induce mild colonic local inflammation but without an obvious impact on the systemic histopathology.

Given that epithelial hyperpermeability is considered prior to the onset of mucosal inflammation,^[^ ^24^ ^]^ we tested the effect of *M. elsdenii* on intestinal barrier function via a FITC‐dextran permeability assay and analyzed the expressions of epithelial tight junction proteins. A higher level of serum FITC‐dextran was observed in *M. elsdenii*‐treated mice (Figure 1G). Tight junction proteins, including Zonulin‐1 (Zo‐1), Zo‐2, Occludin and Claudin 2, showed significantly decreased gene expressions in the colon of the *M. elsdenii* group as determined by qPCR (Figure S1F, Supporting Information), and decreased protein expressions of Zo‐1 and Occludin by IHC (Figure 1H). Collectively, our data demonstrate that *M. elsdenii* alone was insufficient to cause severe colitis and systemic histopathology but impairing colon epithelial homeostasis and barrier function.

### 2.2 *M. elsdenii* Reshapes the Colonic Mucosal Immune Landscape

**Figure 2:**
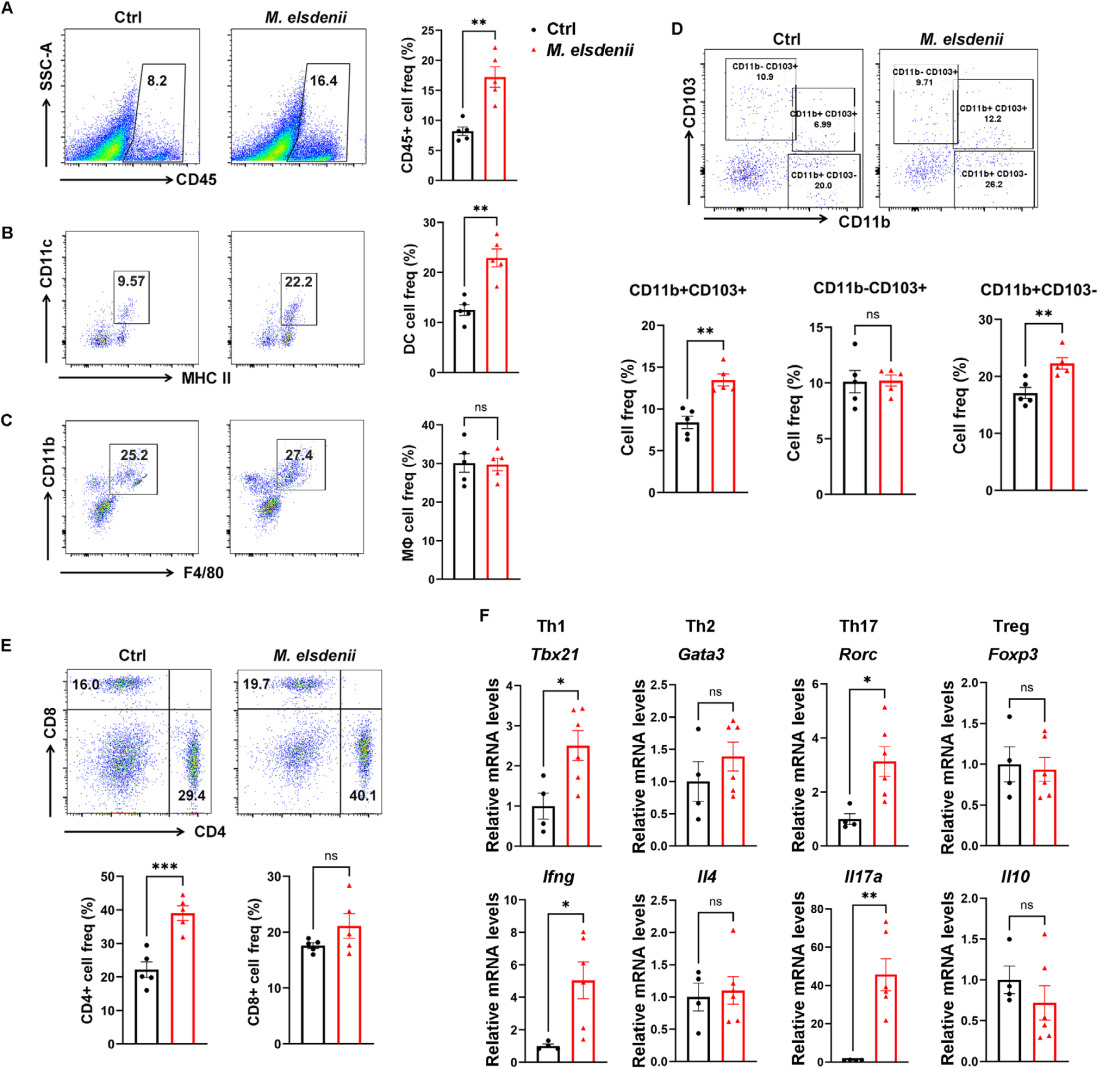
Colonization of *M. elsdenii* remodels the colonic mucosal immune landscape. A) Flow cytometric analysis of colonic lamina propria (LP) CD45+ hematopoietic cells after gavaged with *M. elsdenii* for eight weeks (n = 5/group). B,C) Flow cytometric analysis of CD11c+ MHC II+ dentritic cells B) and CD11b+ F4/80+ macrophages C) in the colonic LP of *M. elsdenii*‐colonized mice (n = 5/group). D) Flow cytometric analysis of DC cell subsets of CD11b+ CD103+ DC, CD11b‐ CD103+ DC and CD11b+ CD103‐ DC (n = 5/group) in the colonic tissue of *M. elsdenii*‐colonized mice. E) Flow cytometric analysis of CD4+ and CD8+ T cells in the colonic LP of *M. elsdenii*‐colonized mice (n = 5/group). F) qPCR analysis of Th1‐, Th2‐, Th17‐, and Treg‐associated transcription factors (*Tbx21*, *Gata3*, *Rorc* and *Foxp3*) and cytokines (*Ifng*, *Il4*, *Il17a* and *Il10*) in colonic tissues of *M. elsdenii*‐colonized mice (n = 4 in control group, n = 6 in *M. elsdenii* group). Each experiment conducted 2–3 replicates. Data presented as mean ± SEM. Statistical analysis was performed with Student's t‐test; ns, not significant, *p < 0.05, **p < 0.01, ***p < 0.001.

An increased intestinal permeability leads to the translocation of bacteria deep into the intestinal tissue, resulting in overactivation of the sub‐epithelial mucosal immune system to defend against intruding pathogens.^[^ ^25^, ^26^ ^]^ Considering that *M. elsdenii* can enhance colonic permeability, we hypothesized that the immune cells resided in colonic lamina propria (LP) could respond to *M. elsdenii*. To explore the effect of *M. elsdenii* on the immune landscape of colonic LP, we tested the colonic immune cell composition of isolated LP cells in *M. elsdenii*‐colonized mice using multicolor flow cytometry analysis. We found an expansion of colonic CD45+ hematopoietic cells and a substantial increase in CD11c+ MHCII+ dendritic cells (DCs) in *M. elsdenii*‐treated SPF and ABX mice compared with PBS‐gavaged controls (**Figures** **2**A,B and S2B,C, Supporting Information), while showing no change in CD11b+ F4/80+ macrophages (Figure 2C and Figure S2D, Supporting Information). It suggests that DCs are more responsive to *M. elsdenii* than macrophages. LPDCs are composed of functionally specialized subsets, distinguished by mutually exclusive surface expression of the integrins CD103 and CD11b and an additional subset expressing both CD103 and CD11b.^[^ ^27^, ^28^ ^]^ Particularly, we found that *M. elsdenii* mainly caused an expansion of CD11b+ CD103+ and CD11b+ CD103‐ DCs in colonic LP (Figure 2D and Figure S2E, Supporting Information). Since CD11b+ DCs serve as inflammatory cells to enhance effector responses to infection,^[^ ^29^ ^]^ our results suggest that *M. elsdenii* could result in a high colonic proinflammatory state.

As to adaptive immune cells, a higher number of CD4+ T cells were observed in colonic LP of *M. elsdenii*‐colonized mice than in PBS‐treated mice, while CD8+ T cells were similar in these two groups (Figure 2E and Figure S2F, Supporting Information). We performed qPCR of colonic tissues to determine the precise initiation of T helper (Th) responses upon *M. elsdenii* colonization. We found an expansion of *Tbx21*/*Ifng* Th1‐ and *Rorc*/*Il17a* Th17‐associated inflammatory responses in *M. elsdenii*‐bearing SPF mice (Figure 2F) while showing no difference in *Gata3*/*Il4* Th2 and *Foxp3*/*Il10* regulatory T (Treg) cells. A similar induction of colonic Th1 and Th17 immune responses was shown in ABX mice colonized with *M. elsdenii* (Figure S2G, Supporting Information). We also found higher expressions of colonic *Il1b* and *Tnfa*, known as carcinogenesis‐involved inflammatory cytokines, in *M. elsdenii*‐treated mice (Figure S2H, Supporting Information).

Given that DC subsets differentially contribute to T cell responses, of which the CD11b+ DC subsets are responsible for instructing Th1 and Th17,^[^ ^27^, ^30^, ^31^ ^]^ we proposed that CD11b+ DCs may mediate *M. elsdenii‐*induced Th1 and Th17 immunity. To determine it, we sorted colonic CD103+ CD11b‐ DCs and CD11b+ DCs and then performed DC subset‐T cell co‐culture system in the presence of *M. elsdenii* or not (Figure S2I, Supporting Information). We showed that CD11b+ DCs instructed Th1 and Th17 cell responses when treated with *M. elsdenii*, demonstrated by the significant increase in the expression of Th1/17 markers (*Tbx21* and *Rorc*) and proinflammatory cytokines (*Ifng* and *Il17a*). However, *M. elsdenii* decreased the gene expression of anti‐inflammatory cytokines *Il4* and *Il10* in CD103+ DCs (Figure S2J, Supporting Information). Together, these data indicated the pro‐inflammatory effects of *M. elsdenii*.

### 2.3 *M. elsdenii* Aggravates Colitis in Germ‐Free Mice

**Figure 3:**
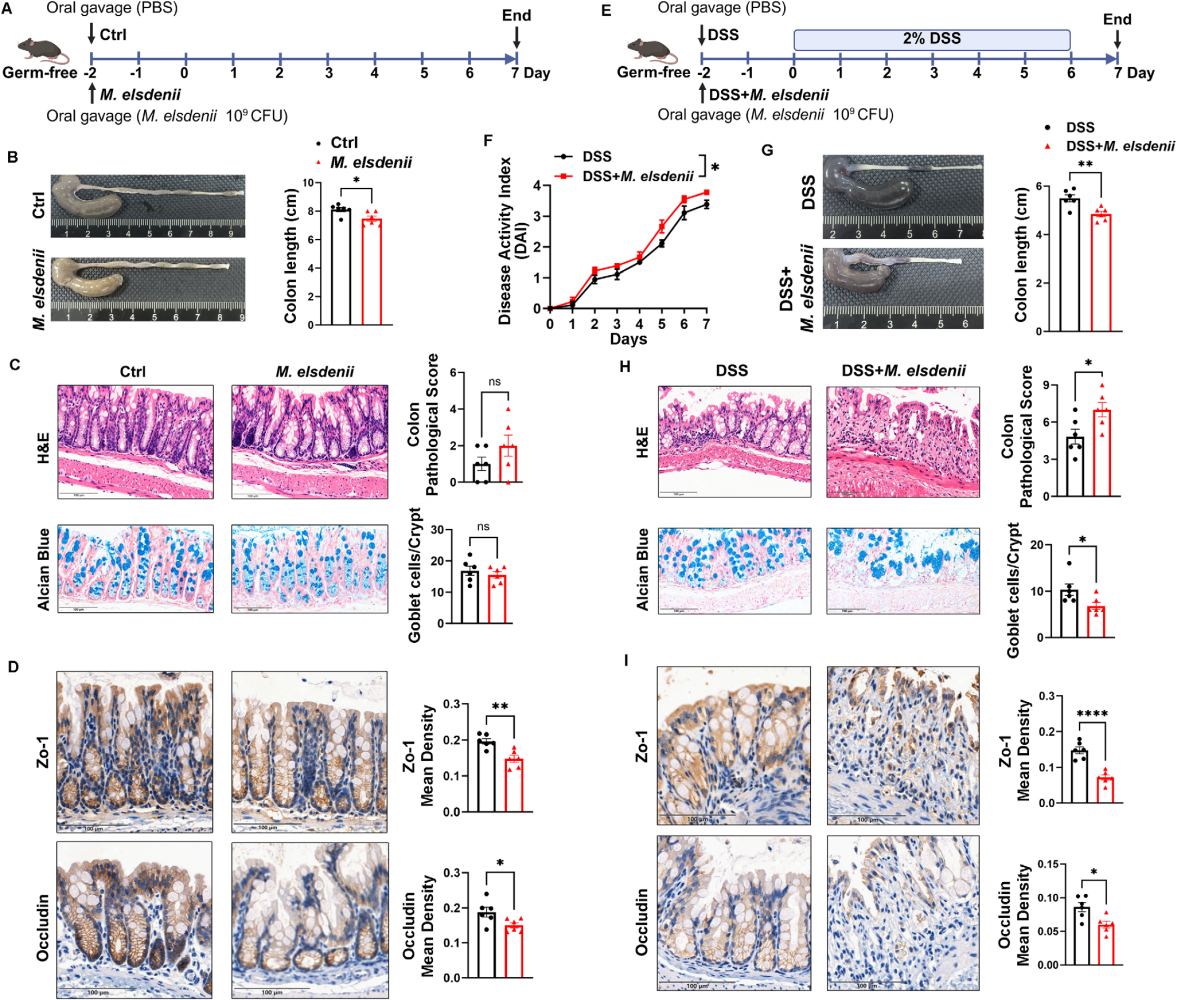
*M. elsdenii* mono‐colonizaiton boosts colonic inflammation and aggravates colitis in germ‐free mice. A) Schematic protocol of *M. elsdenii* colonization in germ free (GF) mice. B) Macroscopic view of the colons and colon length in *M. elsdeni*‐colonized mice (n = 6/group). C) Representative images of H&E (top) and Alcian blue staining (bottom) of colon tissue paraffin‐embedded sections (n = 6/group), scale bar = 100 µm. Histograms showing pathological score and colon goblet cell count of colon tissue sections (n = 6/group). D) IHC staining analysis of colonic Zo‐1 and Occludin. Histograms representing the mean density of Zo‐1 and Occludin, scale bar = 100 µm (n = 6/ group). E) Schematic protocol of *M. elsdenii* colonization in DSS‐induced GF mice (n = 6/group). F) Disease activity index (DAI) score of *M. elsdenii* mono‐colonized DSS mice (n = 6/group). G) Representative macroscopic view of the colons and colonic length in *M. elsdenii*‐colonized DSS mice (n = 6/group). H) Representative images of H&E (top) and Alcian blue staining (bottom) of colon tissue paraffin‐embedded sections (n = 6/group), scale bar = 100 µm. Histograms showing pathological score and colon goblet cell count of colon tissue sections (n = 6/group). I) IHC staining analysis of colonic Zo‐1 and Occludin. Histograms representing the mean density of Zo‐1 and Occludin, scale bar = 100 µm (n = 6/group). Each experiment conducted 2–3 replicates. Data presented as mean ± SEM. Statistical analysis was performed with Student's t test in (B‐D, G‐I) and two‐way ANOVA in (F); ns, not significant, *p < 0.05, **p < 0.01, ****p < 0.0001.

To further determine the role of *M. elsdenii* on colonic physiology and inflammation, we mono‐colonized germ‐free (GF) mice with *M. elsdenii*, and the success colonization of *M. elsdenii* was determined by qRT‐PCR (**Figures** **3**A and S3A, Supporting Information). As expected, *M. elsdenii* resulted in a shortened colon length in GF mice, while having no impact on body weight, spleen index and colon pathological score (Figure 3B and Figure S3B,C, Supporting Information). However, decreased expressions of colonic Zo‐1 and Occludin were noted in *M. elsdenii*‐gaveged GF mice relative to those with PBS (Figure 3D). We also tested the role of *M. elsdenii* in colitis GF model induced by 7 days of dextran sodium sulfate (DSS, Figure 3E). Following DSS, *M. elsdenii* aggravates colitis in GF mice, demonstrated by greater weight loss, higher disease activity score and spleen index, more severe colonic pathological score, shorter colon length and lower expressions of Zo‐1 and Occludin (Figure 3F–I and Figure S3I,J, Supporting Information).

In consistent with the observations in SPF and ABX mice, *M. elsdenii* colonization significantly elevated colonic CD45+ hematopoietic cells, CD11c+ DCs and CD4+ T cells in GF mice whatever given DSS or not (Figure S3D–F,K–M, Supporting Information), along with increased expressions of Th1 and Th17 marker genes (*Tbx21* and *Rorc*) and their effectors (*Ifng* and *Il17a*) (Figure S3G,N, Supporting Information). These results indicated that *M. elsdenii* colonization boosted colonic inflammation and aggravated DSS‐induce colitis in GF mice.

### 2.4 *M. elsdenii* Induces DC Maturation and Activation

**Figure 4:**
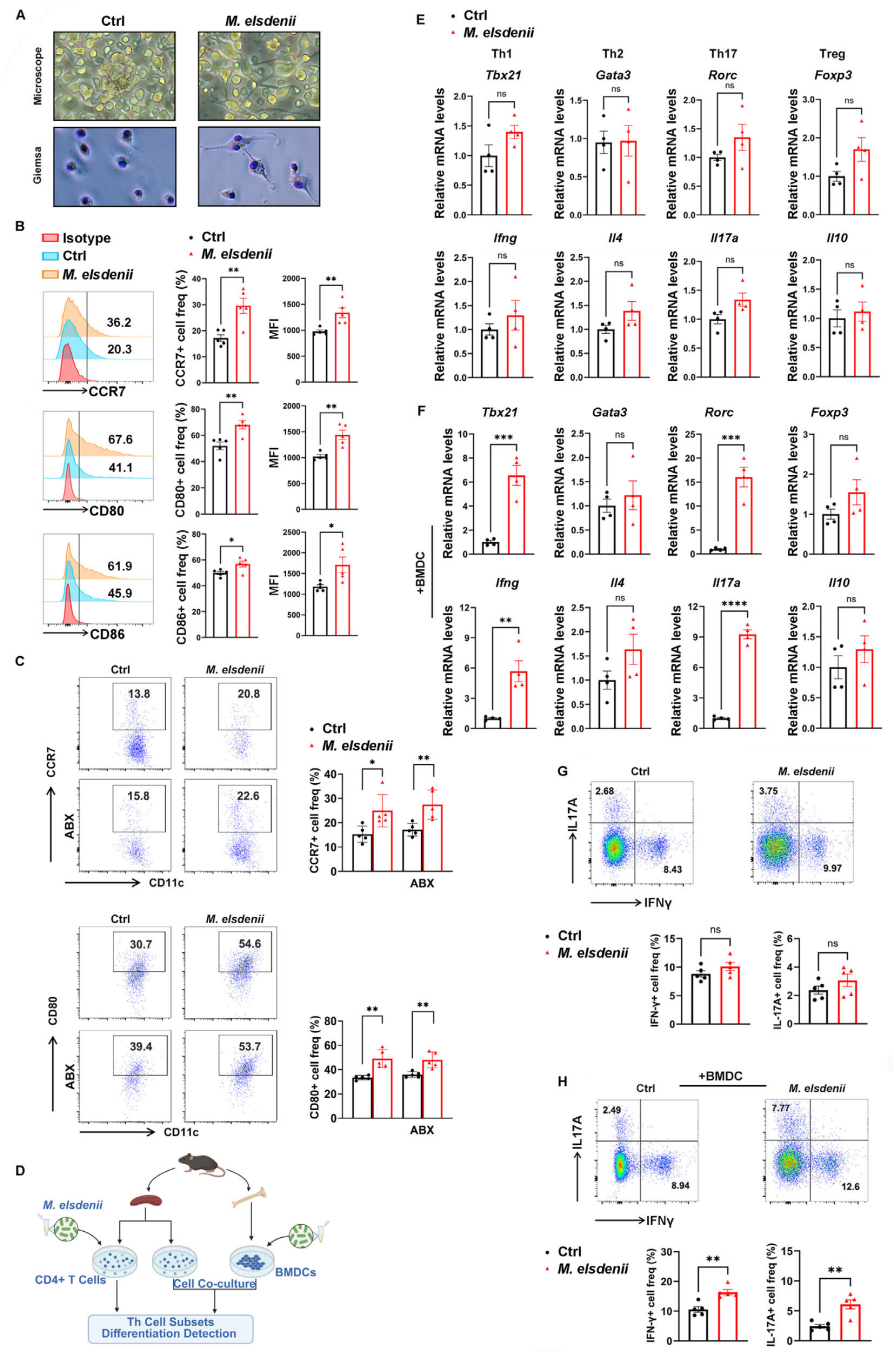
*M. elsdenii* induces Th1 and Th17 immune response through DC cell activation. A) Representative microscopy (top) and Giemsa staining (bottom) morphological images of bone marrow derived DCs (BMDC) treated with PBS or *M. elsdenii*. B) Flow cytometric analysis of CCR7, CD80, and CD86 staining in BMDCs treated with PBS or *M. elsdenii* (n = 5 independent experiments). C) Flow cytometric analysis of CCR7 and CD80 expression in colonic DCs of *M. elsdenii*‐colonized mice (n = 5/group). D) Schematics of *ex vivo* experimental system. E,F) qPCR analysis of Th1‐, Th2‐, Th17‐, and Treg‐associated transcription factors (*Tbx21*, *Gata3*, *Rorc* and *Foxp3*) and cytokines (*Ifng*, *Il4*, *Il17a* and *Il10*) in *M. elsdenii*‐treated CD4+ spleen T cells in the absence (E) or presence (F) of BMDCs (n = 4 independent experiments). G,H) Flow cytometric analysis of *M. elsdenii*‐treated (or not) CD4+ spleen T cells for IFNγ and IL17A production in the absence G) or presence H) of BMDCs (n = 5 independent experiments). Each experiment conducted at least three replicates. Data was shown as mean ± SEM. Statistical analysis was performed with Student's t‐test; ns, not significant, *p < 0.05, **p < 0.01, ***p < 0.001, ****p < 0.0001.

DC cells serve as first‐line defenses against mucosal infections of the gastrointestinal tract and play a central role in priming T cell responses in lymphoid structures.^[^ ^32^ ^]^ The accumulation of colonic LP DCs in *M. elsdenii*‐colonized mice prompted us to focus on the role of *M. elsdenii* in DC cell function, which largely depending on their maturation and activation.^[^ ^33^ ^]^ We isolated bone marrow‐derived DCs (BMDCs) from healthy SPF mice and co‐cultured with *M. elsdenii* (Multiplicity of infection, MOI = 100:1). BMDCs tend to be mature when culturing with *M. elsdenii*, exhibiting the characteristics of fine dendrites and large cytoplasm (**Figure** **4**A). However, those cells without *M. elsdenii* treatment showed smoother cell membranes, characterized as immature nonstimulated DCs. As expected, flow cytometry showed that *M. elsdenii* upregulated expressions of chemokine receptor CCR7, costimulatory receptors CD80 and CD86, the DC maturation and activation markers (Figure 4B and Figure S4A, Supporting Information). Likewise, we found elevated surface expression of CCR7 and CD80 among colonic DCs in mice colonized with *M. elsdenii* (Figure 4C).

To determine whether *M. elsdenii* directly affects Th responses or dependent on DC cell activation, we isolated spleen CD4+ T cells and established a cell coculture system with *M. elsdenii* pretreated BMDCs (Figure 4D). No significant difference of Th subsets was found in T cells with and without *M. elsdenii* treatment (Figure 4E). In the presence of BMDCs, however, increased Th1 and Th17 responses were noteworthy upon treatment with *M. elsdenii* (Figure 4F). Consistently, no difference in Th1‐cytokine IFNγ+ and Th17‐cytokine IL17A+ cells was observed in the absence of BMDCs (Figure 4G and Figure S4B, Supporting Information), while the levels of IFNγ+Th1 and IL17A+Th17 cells were significantly induced in the coculture system with *M. elsdenii* pretreated BMDCs (Figure 4H). These results revealed that *M. elsdenii*‐triggered Th1 and Th17 inflammatory responses depend on the maturation and activation of DCs.

### 2.5 *M. elsdenii* Modulates DC Functions through TLR4/NF‐κB/IRF4 Signaling

**Figure 5:**
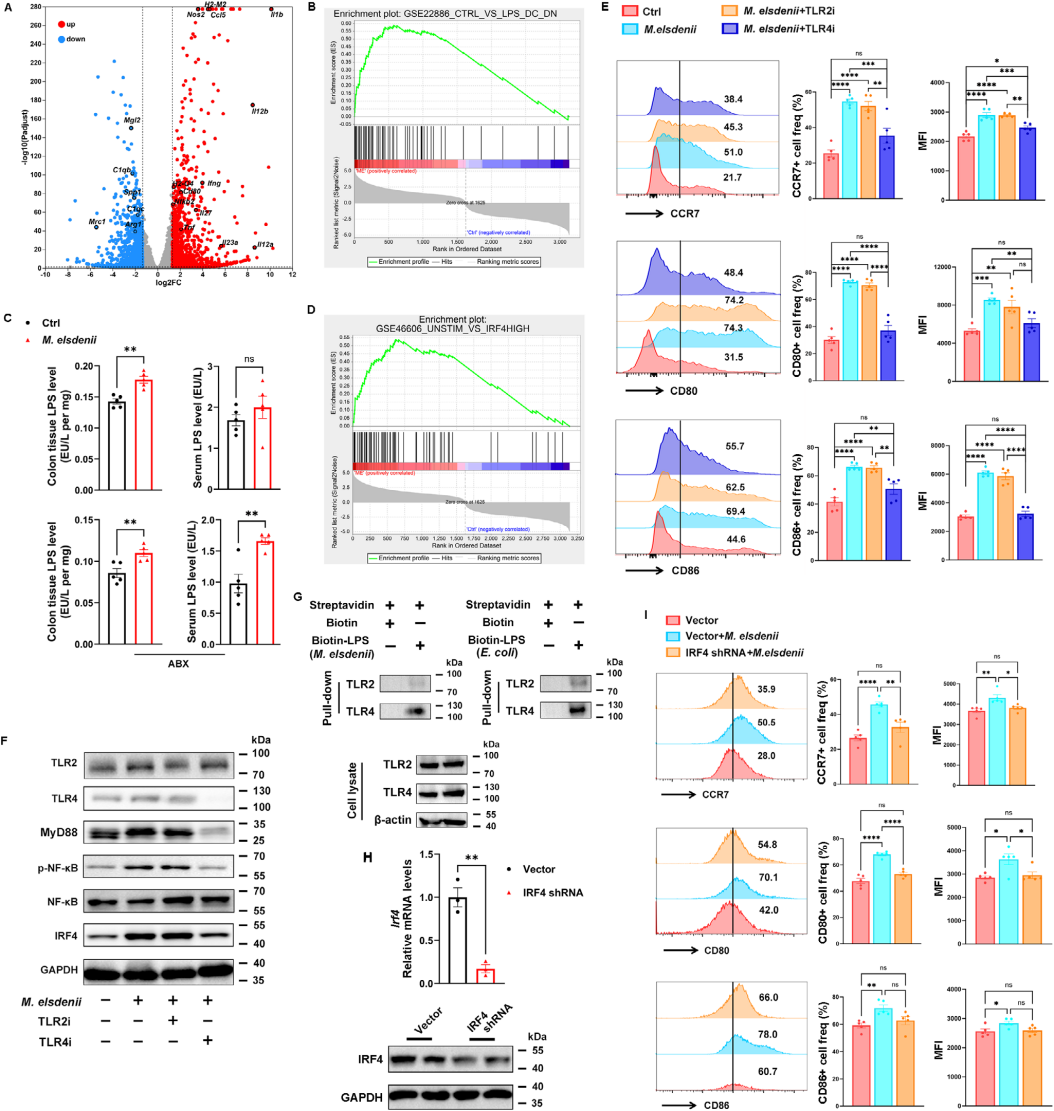
*M. elsdenii*‐mediated DC activation depends on TLR4/NF‐κB/IRF4 pathway. A) Volcano plot of RNA sequencing dataset showing differential expressed genes (DEGs) of BMDCs infected with *M. elsdenii* compared with PBS treated BMDCs (*p*<0.05, fold‐change [FC] R 2.0) derived from biological triplicates. Red: upregulated, blue: downregulated. B) Gene set enrichment analysis (GSEA) showing the enrichment of LPS‐treatment signature. C) Colonic tissue supernatants and serum LPS levels were determined using ELISA in *M. elsdenii*‐treated SPF mice (top) or ABX‐mice (bottom) (n = 5/group). D) GSEA showing the enrichment of IRF4‐high signature. E) Flow cytometric analysis of CCR7, CD80 and CD86 expression in *M. elsdenii*‐treated BMDCs with and without TLR2/4 antagonist (n = 5 independent experiments). F) Western blot analysis of key proteins of TLR4/NF‐κB/IRF4 pathway in TLR2/4 antagonist treated BMDCs. G) Pull‐down analysis of BMDC lysates incubated with biotin‐labeled LPS derived from *M. elsdenii* or *E. coli*. H) qPCR analysis of *Irf4* mRNA levels (top) and western blot analysis of IRF4 protein levels (bottom) in BMDCs transfected with IRF4 shRNA or vector (n = 5 independent experiments). I) Flow cytometric analysis of CCR7, CD80 and CD86 expressions in *M. elsdenii* or PBS treated BMDCs transfected with IRF4 shRNA or vector (n = 5 independent experiments). Each experiment conducted at least three replicates. Data presented as mean ± SEM. Statistical analysis was performed with Student's *t* test C, H), one‐way ANOVA E, I); ns, not significant, *p < 0.05, **p < 0.01, ***p < 0.001, ****p < 0.0001.

To investigate the mechanism by which *M. elsdenii* affects DC function, we sequenced BMDC RNAs with and without *M. elsdenii* treatment. A subset of genes involved in immune activation, inflammation and antigen presentation, including *Il1b*, *Ifng*, *Il23*, *Il12*, *Cd40*, *Icos*, *Cd80*, *H2‐M2* and *H2‐K2*, were increased by *M. elsdenii*, whereas the species downregulated immunosuppressive genes (*Arg1*, *Mrc1*, *Cd163*) (**Figures** **5**A and S5A, Supporting Information). Gene set enrichment analysis (GSEA) showed that lipopolysaccharide (LPS)‐activated gene set was enriched in *M. elsdenii*‐treated BMDCs (Figure 5B). Considering that *Megasphaera* is Gram‐negative bacteria and LPS is a well‐characterized integral component of the outer membrane of Gram‐negative bacteria to initiate innate immune responses mediated by DCs,^[^ ^34^ ^]^ we assessed colonic and serum LPS concentrations in mice infected with *M. elsdenii*. Elevated LPS levels were shown in the colonic tissue and serum of *M. elsdenii*‐colonized mice (Figure 5C).

As LPS is a key component that promotes DC activation, we speculated that the LPS of *M. elsdenii* may drive DC activation. We purified LPS from *M. elsdenii* and then treated BMDCs with *M. elsdenii*‐derived LPS or *E. coli*‐derived LPS to clear the direct interaction of *M. elsdenii*‐derived LPS with DCs. Results showed that LPS from *M. elsdenii* can induce BMDCs activation with increased expression of CD80, CD86 and CCR7, which capability to promote DC activation was comparable to that of *E. coli*‐derived LPS (Figure S5B, Supporting Information).

Enrichment analysis of the KEGG pathway and GO term showed that the Toll‐like receptor (TLR) signaling pathway and NF‐κB signaling pathway in BMDCs were notably upregulated by *M. elsdenii* (Figure S5C,D, Supporting Information). Previous studies have demonstrated a central role for interferon regulatory factor 4 (IRF4) in regulating the DC maturation and activation program,^[^ ^35^ ^]^ and IRF4‐dependent DCs are required for inducing Th cell responses with an emphasis on stimulating intestinal Th17 cell differentiation.^[^ ^36^, ^37^, ^38^ ^]^ Identically, the enrichment score showed an IRF4‐high signature in *M. elsdenii*‐treated BMDCs (Figure 5D). Since TLR2 and TLR4 are the primary recognition receptors in microbial recognition, we assessed the effect of *M. elsdenii* on BMDCs using selective TLR2 or TLR4 antagonist. As shown in Figure 5E, only the TLR4 antagonist can abolish the up‐regulated expression of CCR7, CD80 and CD86 in *M. elsdenii*‐treated BMDCs. Hence, we speculate that *M. elsdenii* may trigger DC maturation and activation via LPS/TLR4/NF‐κB/IRF4 signaling. We further confirmed that protein expression levels of TLR4/MyD88/NF‐κB cascades, concomitant with the induction of IRF4 were increased in BMDCs treated with *M. elsdenii*‐derived LPS (Figure S5E, Supporting Information). Antagonizing TLR4 but not TLR2 can block *M. elsdenii*‐activated NF‐κB/IRF4 pathway (Figure 5F).

To validate the interaction between the TLR receptors and *M. elsdenii* LPS, we used biotin‐labeled LPS and performed LPS pull‐down to precipitate its binding proteins. *M. elsdenii*‐derived LPS was strongly capable to bind with TLR4, but a much weaker binding to TLR2 (Figure 5G). To confirm the role of IRF4, we made a knockdown assay with IRF4 shRNA (Figure 5H). Knockdown of IRF4 partially abrogated the increased CCR7, CD80 and CD86 expression evoked by *M. elsdenii* (Figure 5I), with 64.9%/54.45%/69.36% reduction in CCR7/CD80/CD86 expression post‐IRF4 knockdown respectively. We further performed chromatin immunoprecipitation (ChIP) qPCR to detect its potential target genes and showed that IRF4 could bind to target genes *Il1b*, *Il23*, *Cd80* and *Cd86* (Figure S5F, Supporting Information). These results collectively indicated that *M. elsdenii* promotes DC maturation and activation through TLR4 ligation, leading to the activation of NF‐κB and IRF4 and subsequent induction of pro‐inflammatory cytokines and co‐stimulatory molecules, which are required for priming Th‐cell responses.

### 2.6 *M. elsdenii* Promotes Colitis‐Associated Tumorigenesis

**Figure 6:**
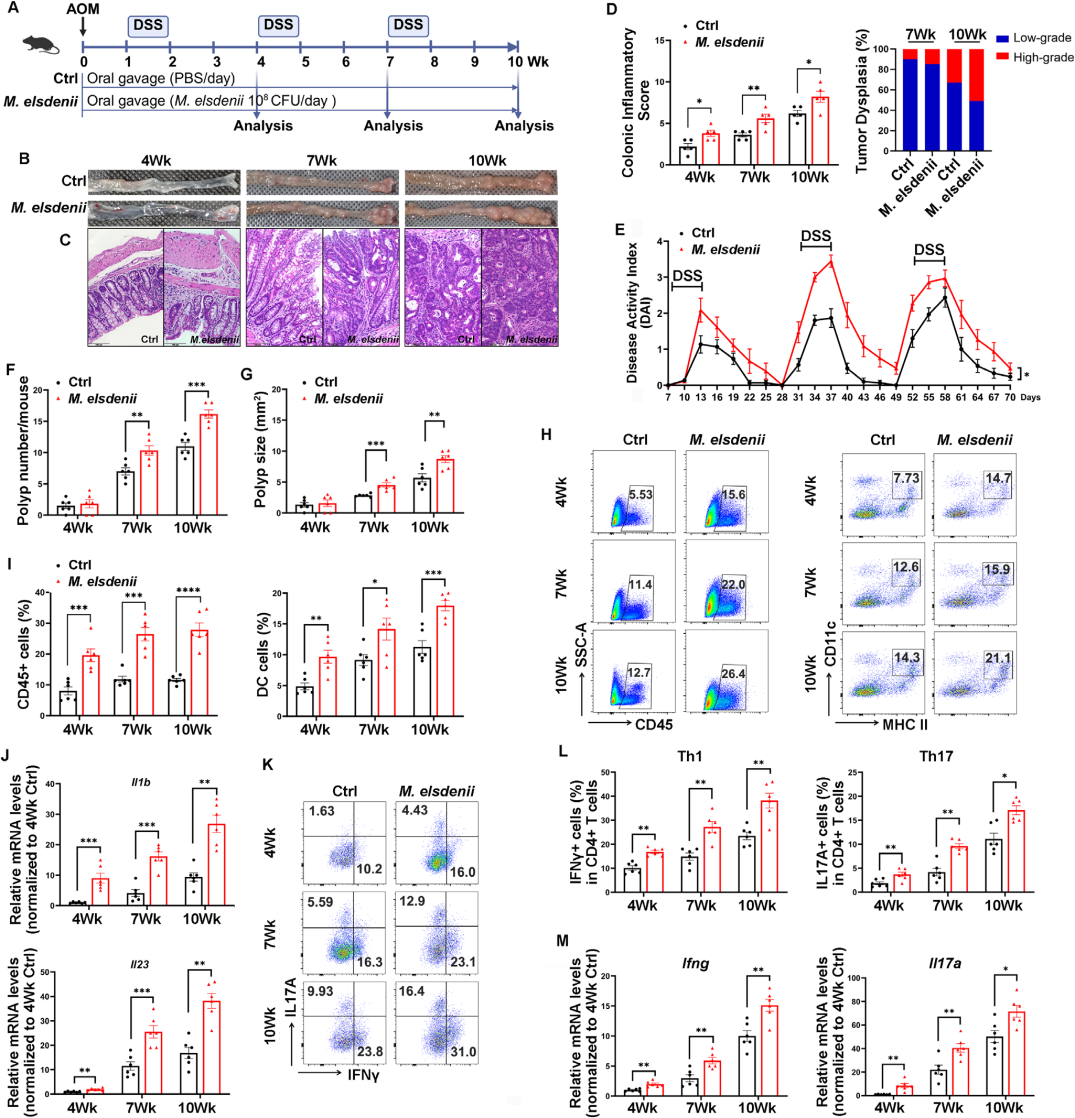
*M. elsdenii* promotes colitis‐associated tumorigenesis. A) Schematic of in vivo experiment design and timeline of AOM/DSS model. B) Representative macroscopic view of the colons and polyps throughout disease progression (week4/7/10) in *M. elsdenii*‐treated AOM/DSS mice. C) Representative H&E images of colonic tissue sections from AOM/DSS mice administrated with *M. elsdenii*, scale bar = 100 µm. D) Histological scoring of colonic inflammatory pathology and dysplasia of tumors (week4/7/10: n = 5/group). E) DAI score of *M. elsdenii*‐treated AOM/DSS mice (week4/7/10: n = 6‐8/group). F, G) Polyp numbers F) and sizes G) in *M. elsdenii*‐treated AOM/DSS mice (week4/7/10: n = 6/group). H, I) Flow cytometric analysis of CD45+ hematopoietic cells and CD11c+ MHC II+ DCs frequency in colonic LP of *M. elsdenii*‐treated AOM/DSS mice (week4/7/10: n = 6/group). J) qPCR analysis of DC‐activated proinflammatory genes *Il1b* and *Il23* in the colon tissue of *M. elsdenii*‐colonized AOM/DSS mice (week4/7/10: n = 6/group). K, L) Flow cytometric analysis of IFNγ+ and IL17A+ cells in CD4+ T cells in the colonic LP of *M. elsdenii*‐treated AOM/DSS mice (week4/7/10: n = 6/group). M) qPCR analysis of *Tbx21*/*Ifng* and *Rorc*/*Il17a* mRNA levels in the colonic tissues of *M. elsdenii*‐colonized AOM/DSS mice (week4/7/10: n = 6/group). Each experiment conducted 2–3 replicates. Data presented as mean ± SEM. Statistical analysis was performed with Student's *t*‐test D, F, G, I–M), two‐way ANOVA E); *p < 0.05, **p < 0.01, ***p < 0.001, ****p < 0.0001.

To clarify the impact of *M. elsdenii* on colitis‐associated tumorigenesis, we colonized the species to colitis‐associated cancer (CAC) mice receiving AOM (12.5 mg kg^−1^), followed by three cycles of 2.5% DSS (**Figure** **6**A). On the day of AOM administration, mice were orally gavaged with *M. elsdenii* (10^8^ CFU per day) or PBS until sacrifice. Mice were analyzed at weeks 4, 7 and 10 to mimic the sequential processes of CAC initiation from inflammation and dysplasia to adenocarcinoma.^[^ ^39^ ^]^ With disease progression, strikingly increased number and size of macroscopic polyps were observed in *M. elsdenii*‐treated mice compared with controls (Figure 6B,F,G). *M. elsdenii* also worsened histological colitis, as evidenced by increased mucosal hyperplasia and submucosal immune cell infiltration (Figure 6C,D). *M. elsdenii*‐colonized mice exhibited a higher DAI score, greater weight loss and enlarged spleen at the week 10 endpoint (Figure 6E and Figure S6A,B, Supporting Information).

Flow cytometry data showed that *M. elsdenii* caused an expansion of hematopoietic cells and DCs in the colonic LP of CAC mice (Figure 6H,I), similar to its effects observed in *M. elsdenii*‐infected normal mice. The transcript of DC‐activated proinflammatory genes (*Il1b* and *Il23*) was gradually increased in the colon of mice with the development of CAC and showed much higher levels in *M. elsdenii*‐gavaged mice (Figure 6J). Also, *M. elsdenii* colonization induced more severe Th1 and Th17 responses in the colonic mucosa (Figure 6K–M). However, there was no difference in macrophages and CD8+ T cells in CAC mice between *M. elsdenii* and PBS groups (Figure S6D,E, Supporting Information). We observed an expansion of immunosuppressive Treg responses in CAC mice with *M. elsdenii* colonization at week 7 and week 10 (Figure S6F, Supporting Information), which may be due to a feedback loop of a compensatory Treg response to the hyper‐inflammation stimulus.^[^ ^40^ ^]^ We also assessed the TLR4 signaling pathway in colonic tissues using western blot, and found that the expression levels of TLR4, MyD88, p‐NF‐κB and IRF4 were significantly increased in *M. elsdenii*‐gavaged AOM/DSS mice compared to controls (Figure S6G, Supporting Information), indicating TLR4/NF‐𝜅B/IRF4 pathway activated by *M. elsdenii* in the CAC model. These data confirm that the inflammatory milieu created by *M. elsdenii* increases the susceptibility of developing CAC.

### 2.7 *M. elsdenii* has No Effect on Colonic Inflammation and CAC Tumorigenesis in *Tlr4*‐Deficient Mice

**Figure 7:**
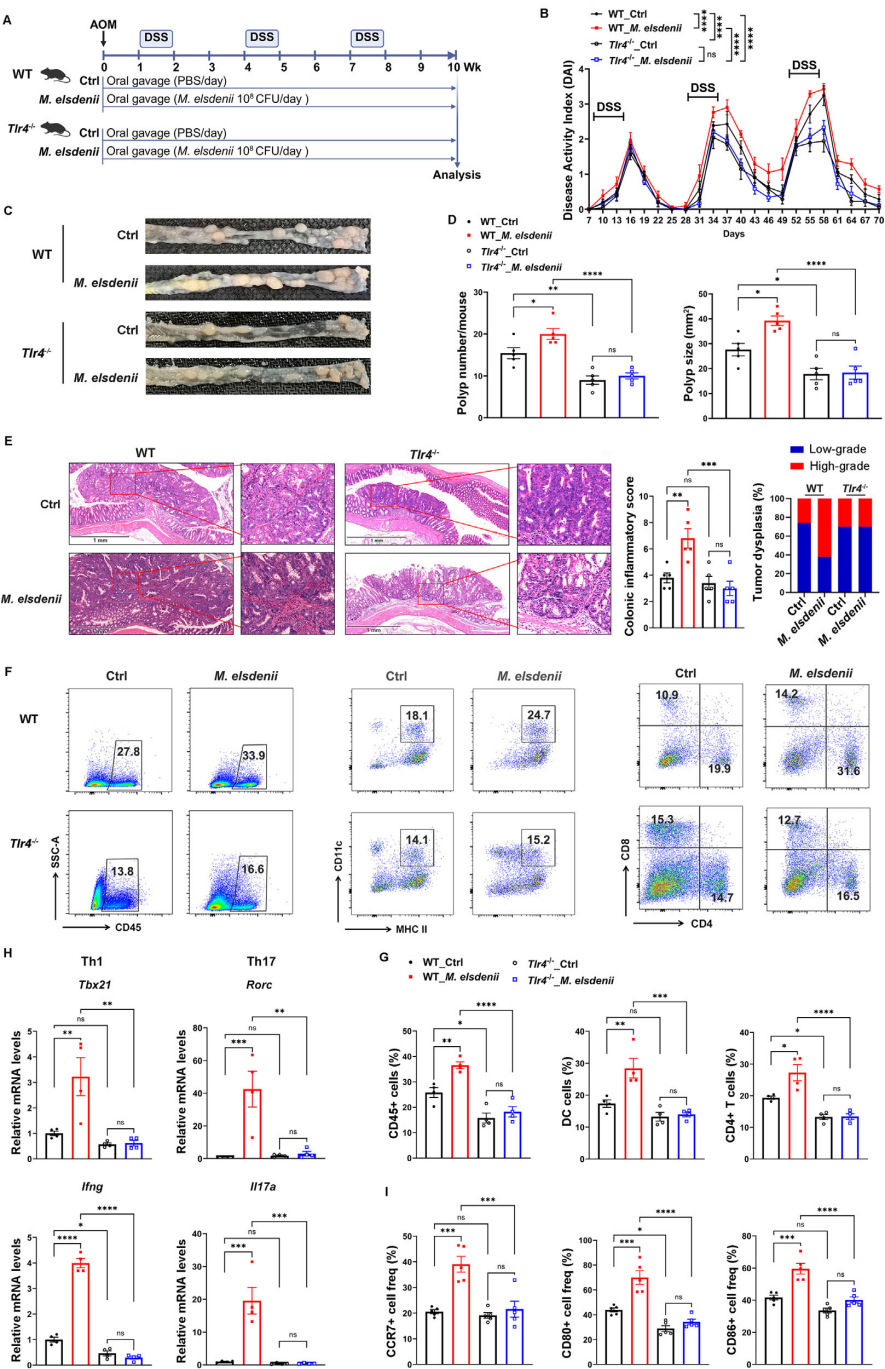
*M. elsdenii* has no impact on inflammation‐related tumorigenesis in *Tlr4*‐deficient mice. A) Schematic of in vivo experiment design of *M. elsdenii*‐colonized WT and *Tlr4* ^−/−^ AOM/DSS model. B) DAI score of mice (n = 7–8/group). C) Representative macroscopic view of the colons and polyps in *M. elsdenii*‐colonized WT and *Tlr4* ^−/−^ AOM/DSS mice. D) Polyp numbers (left) and size (right) in *M. elsdenii*‐colonized WT and *Tlr4* ^−/−^ AOM/DSS mice (n = 5/group). E) Representative H&E images of colonic tissue sections (Left, scale bar = 1 mm) and histological scoring of colonic inflammatory pathology and dysplasia of tumors (right). F, G) Flow cytometric analysis of CD45+ hematopoietic cells, CD11c+MHCII+ DCs, and CD4+ T cells frequency in colonic LP of *M. elsdenii*‐colonized WT and *Tlr4* ^−/−^ AOM/DSS mice (n = 4/group). H) qPCR analysis of Th1‐ and Th17‐associated transcription factors (*Tbx21* and *Rorc*) and cytokines (*Ifng* and *Il17a*) in the colon tissue (*n* = 4/group). I) Flow cytometric analysis of CCR7, CD80, and CD86 expression in *Tlr4*‐deficient or WT BMDCs (*n* = 5 independent experiments). Each experiment conducted at least three replicates. Data presented as mean ± SEM. Statistical analysis was performed with two‐way ANOVA B), one‐way ANOVA (D, E, G‐I); *p < 0.05, **p < 0.01, ***p < 0.001, ****p < 0.0001.

To testify *M. elsdenii*‐associated DC activation and tumorigenesis in mouse CAC model is TLR4‐dependent, AOM/DSS and *M. elsdenii* treated wide‐type (WT) and *Tlr4* ^−/‐^ mice were used (**Figure** **7**A). We firstly showed that *M. elsdenii* could colonize in *Tlr4* ^−/‐^ mice (Figure S7A, Supporting Information). Following AOM/DSS treatment, *Tlr4* ^−/‐^ mice exhibited lower weight loss and DAI score, also showed decreased tumor number and size versus WT mice (Figure 7B–D and Figure S7B, Supporting Information). Of note, *M. elsdenii*‐colonized *Tlr4* ^−/‐^ mice displayed similar weight loss and DAI score while exhibiting identical tumor number and size during the AOM/DSS cycle as that in PBS‐treated *Tlr4* ^−/‐^ mice (Figure 7B–D and Figure S7B, Supporting Information). The colonic pathology in *Tlr4* ^−/‐^ mice was significantly lower than that in WT mice but showed no difference in histologic pathology in *Tlr4* ^−/‐^ mice with and without *M. elsdenii* colonization (Figure 7E). Flow cytometry results showed that the number of hematopoietic cells and DC cells was dramatically reduced in *Tlr4* ^−/‐^ mice, accompanied by reduced Th1 and Th17 inflammation, as compared with WT mice. *M. elsdenii* could neither stimulate hematopoietic cells and DC cells nor induce Th1 and Th17 responses in *Tlr4* ^−/−^mice (Figure 7F–H). *M. elsdenii* resulted in significant upregulation of DC activation‐associated proinflammatory genes (*Il1b* and *Il23*) in WT mice while having no impact in *Tlr4* ^−/−^ mice (Figure S7C, Supporting Information). No difference in macrophages and CD8+ T cells, and Th2 and Treg responses, was observed between tumor‐bearing WT and *Tlr4* ^−/−^mice, regardless of the presence of *M. elsdenii* (Figure S7E,F, Supporting Information). We further isolated BMDCs from *Tlr4* ^−/−^mice and stimulated them *ex vivo* with or without *M. elsdenii* to confirm the role of *M. elsdenii* on DC activation under the condition of *Tlr4* deletion. As expected, *Tlr4*‐deficiency eliminated the increased CCR7, CD80 and CD86 expression triggered by *M. elsdenii* (Figure 7I). These results imply that *M. elsdenii* does not affect colonic inflammation and CAC tumorigenesis under a *Tlr4*‐deficient condition.

### 2.8 Transferring *M. elsdenii*‐Enriched Human Microbiota Promotes Murine CAC Development

**Figure 8:**
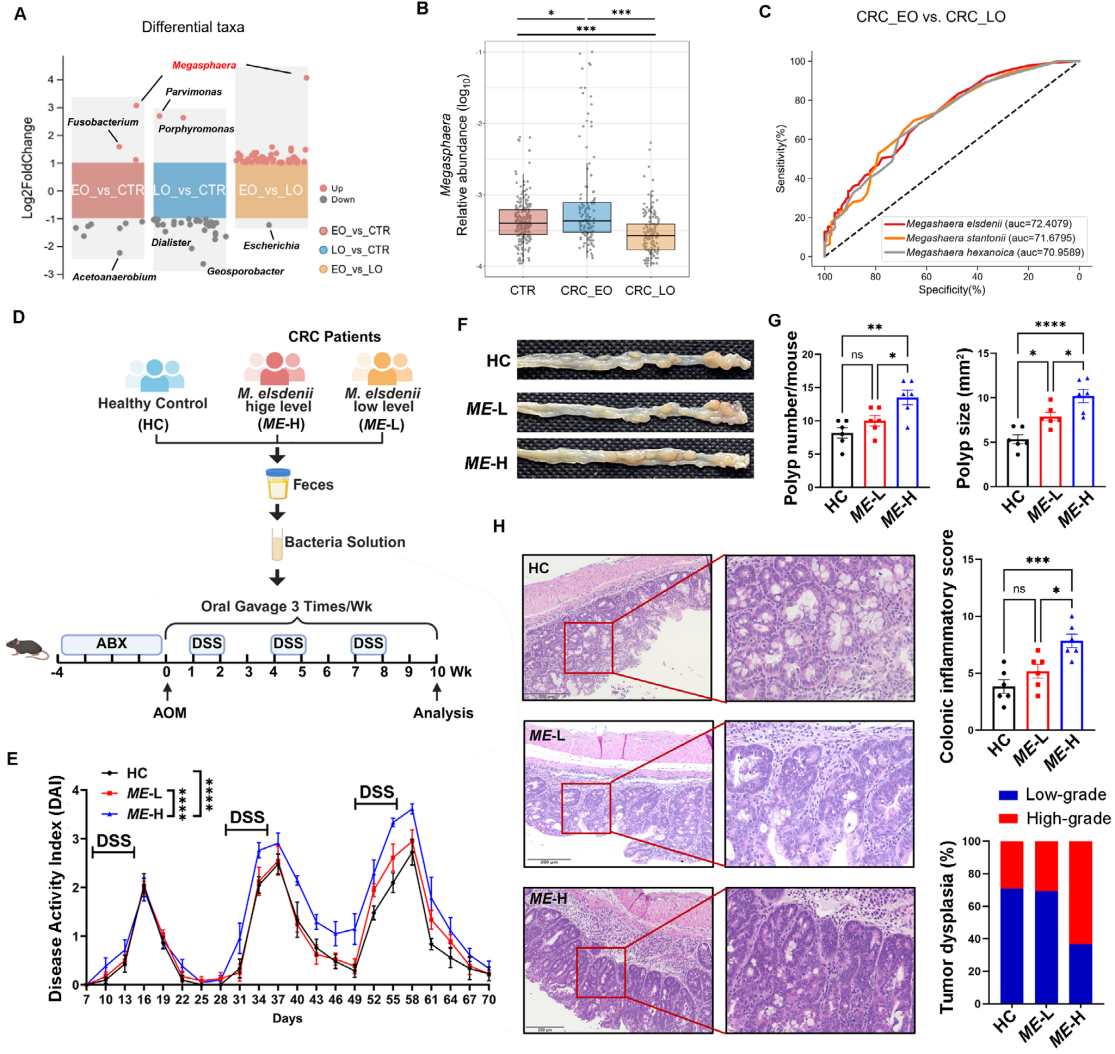
Transplanting of *M. elsdenii*‐abundant fecal microbiota from CRC patients accelerates colitis‐associated tumorigenesis. A) Differential genera in fecal microbiota community among health controls (HC), early‐onset (EO‐CRC) and late‐onset (LO‐CRC) groups. B) Relative abundance of *Megasphaera* among HC, EO‐CRC and LO‐CRC subjects. C) Receiver‐operating characteristic (ROC) curves for the discrimination of EO‐CRC away from LO‐CRC using three *Megasphaera* species. D) Schematic of fecal microbiota transplanting (FMT) experimental protocol. AOM/DSS mice transplanted with fecal microbiota derived from healthy control (HC), *M. elsdenii*‐low level (*ME*‐L) and *M. elsdenii*‐high level (*ME*‐H) donors from recruited CRC patients. E) DAI score of mice (n = 7‐8/group). F) Representative macroscopic view of the colons and polyps. G) Polyp numbers (left) and size (right) in mice (n = 6/group). H) Representative H&E images of colonic tissue sections (left, Scale bar = 200 µm) and histological scoring of colonic inflammatory pathology and dysplasia of tumors (right). Each experiment conducted 2–3 replicates. Data presented as mean ± SEM. Statistical analysis was performed with the Kruskal–Wallis test A, B), one‐way ANOVA G, H), and two‐way ANOVA E); *p < 0.05, **p < 0.01, ***p < 0.001, ****p < 0.0001.

We further analyzed our in‐house fecal metagenomic data from 222 health controls (HC), 125 early‐onset (EO‐CRC, <50 years old) and 151 late‐onset (LO‐CRC, ≥50 years old) CRC patients with a particular emphasis on the level of *Megasphaera*. The clinical characteristics of recruited subjects are summarized in Table S4 (Supporting Information). The positive rate of *Megasphaera* is 100% in this cohort,^[^ ^41^ ^]^ and a significantly high abundance of *Megasphaera* was shown in EO‐CRC compared with HC or LO‐CRC (**Figure** **8**A,B and Table S5, Supporting Information). Three *Megasphaera* species (*M. elsdenii*, *M. stantonii* and *M. hexanoics*) identified in the cohort were found to be sufficient to distinguish EO‐CRC away from LO‐CRC, which area values under receiver operating characteristic (ROC) curve are 0.724, 0.717 and 0.709 separately (Figure 8C). Considering EO‐CRC is more closely associated with inflammatory risk factors (e.g., smoking, IBD, obesity, diabetes) than LO‐CRC,^[^ ^42^ ^]^ our results indicate that *Megasphaera* species are potential biomarkers for EO‐CRC. A *Megasphaera* also showed positive correlation with certain pathogens and lactate or volatile fatty acid‐producing bacteria (Figure S8A, Supporting Information), suggesting it may interact with other microbes to synergistically shape a tumor‐favorable gut ecological niche since we observed that *M. elsdenii* alone was insufficient to cause severe colitis and systemic histopathology.

To confirm a causal relationship between *M. elsdenii*‐abundant human gut microbiota and colorectal tumorigenesis, we performed fecal microbiota transplantation (FMT) in CAC mice by selecting *M. elsdenii*‐low level (*ME*‐L) and *M. elsdenii*‐high level (*ME*‐H) donors from recruited CRC patients (Figure 8D). The abundances of *Megasphaera* and *M. elsdenii* were analyzed by fecal metagenomic sequencing and confirmed by qPCR (Figure S8B,C, Supporting Information). We further analyzed the donors’ fecal microbiota communities with metagenomic analysis. OTU‐based results showed that *ME*‐H and *ME*‐L CRC donors present more similar microbiota profiles than healthy donors, of which *ME*‐H microbiota was enriched with *Dialister*, *Corynebacterium*, *Lactobacillus* and *Erysipelatoclostridium* in addition to *Megasphaera*, while *ME*‐L microbiota is highly abundant with *Parvimonas* and *Peptostreptococcus* (Figure S8D,E, Supporting Information). We also performed qPCR of mouse fecal samples to monitor the abundance of *M. elsdenii* following FMT (Figure S8F, Supporting Information). We observed deteriorated weight loss and DAI score in the *ME*‐H group relative to the HC and *ME*‐H groups (Figure 8E; Figure S8G, Supporting Information). Tumor numbers and tumor size were higher in *ME*‐H mice than in HC and *ME*‐L mice (Figure 8F,G, Supporting Information). Similar to previous observations in *M. elsdenii*‐colonized mice, FMT of *ME*‐H gut microbiota of CRC donors led to more severe pathological changes (Figure 8H), along with higher levels of DC activation‐related inflammatory cytokines *Il1b* and *Il23*, increased infiltration of CD11c+ DC number, and elevated Th1 and Th17 responses (Figure S8H–J, Supporting Information). Moreover, colonic proteins related to the TLR4/NF‐𝜅B/IRF4 signaling pathway were also upregulated in *ME*‐H mice (Figure S8K, Supporting Information). These results reveal a contributive role of *M. elsdenii*‐enriched human microbiota in enhancing colitis‐associated tumor evolution.

## 3 Discussion

In this study, we reported a new role of *Megasphaera* spp. in boosting colonic inflammation and colitis‐associated tumorigenesis by activating DC‐mediated Th1 and Th17 immune responses. It implies that *Megasphaera* species have the potential to be a discriminative biomarker for IBD and early‐onset CRC.

Intriguingly, we observed *Megasphaera* is more prevalent in Asian (China, Japan, India) cohorts. Also, *Megasphaera* has been documented to be abundant in Bengali and Tripura populations,^[^ ^43^ ^]^ which suggests that this genus might be characteristic of Asian populations. Given that a high intake of fermented food, dairy products and carbohydrate‐rich diets is associated with the predominance of *Megasphaera*,^[^ ^44^, ^45^ ^]^ we suppose that the dietary pattern of Asians may be a factor in forming such geographic distribution. However, it remains need further large‐scale, global and multiethnic dataset validation.

Though there remains a controversial association between the bacteria and the health status of the host, it is consensus that the overabundance of *Megasphaera* has been particularly linked with diverse inflammation‐related conditions, such as obesity, prediabetes, rheumatoid arthritis, IBD and CRC.^[^ ^15^, ^16^, ^17^ ^]^ These observations suggest an impact of *Megasphaera* species on dysregulating the immune response of the host, which is also supported by our data that *Megasphaera* promoted colitis‐associated tumorigenesis by exacerbating inflammation. Current insights on the function of the bacteria remain limited, and more in‐depth investigations on clarifying the role of *Megasphaera* species in the (patho)physiology are warranted.

Of note, FMT of *M. elsdenii*‐enriched human fecal microbiota cannot rule out the confounding effects of other bacteria other than *Megasphaera* in enhancing CAC. The metagenomic analysis of *Megasphaera*‐high abundant donors’ microbiota shows no difference in well‐proven CRC pathogens among the three donor groups, such as *Fusobacterium*, *Enterococcus*, and *Salmonella*, which partially rule out the confounding effects of specific CRC pathogens on recipient colon tumorigenesis but not excluding the potential impact of proinflammatory bacteria (e.g., *Escherichia‐Shigella*). Studies of rumen microbial community or cultural experiments support that *Megasphaera*, showing high lactate‐utilizing capability, could be promoted by lactate‐producing bacteria.^[^ ^46^, ^47^ ^]^ *Megasphaera* is also considered to contribute to the gut microbial ecosystem by supplying ammonia and volatile fatty acids that are required by many species of bacteria.^[^ ^48^, ^49^ ^]^ Existing evidence suggests that it might interact with other microbes to synergistically shape inflammation/tumor‐favorable gut ecological niche. Taken together, *M. elsdenii* and its enriched microbiota are capable of boosting colon inflammation and thus promoting colitis‐associated tumorigenesis.

We provide reliable evidence to uncover the previously unknown effect of *Megasphaera spp*. on host colonic mucosa, in which the species induces gut epithelial barrier instability and increase Th1 and Th17 cell responses. Although we did not directly test the role of Th1/Th17 cells in CAC using neutralizing anti‐ IL17A or anti‐IFNγ antibodies, which is one of the limitations of this study, it is well established that Th1/Th17 cell response is a hallmark of colonic inflammation and CAC. In fact, the major role for a pathogenic Th1/Th17 cell response was suggested by showing that anti‐IFNγ antibody/anti‐IL17A antibody treatment significantly attenuated intestinal inflammation, led to fewer and smaller tumors in AOM/DSS mice.^[^ ^50^, ^51^ ^]^ Abrogation of Th1 response using Smad4^TKO^/IFNγ^KO^ mice or abrogation of Th17 response using *Il17a* ^−/−^ mice showed a significantly reduced incidence and progression of CAC, which also suggested the role of IFNγ and IL17a in promoting CAC.^[^ ^52^, ^53^ ^]^ In addition to *Megasphaera* studied by us, certain bacteria abundantly enriched in IBD and CRC patients, such as adherent‐invasive *Escherichia coli*, enterotoxigenic *Bacteroides fragilis* and *Fusobacterium nucleatum*, have also been reported to increase Th17 immune response during bacteria‐driven colon inflammation or tumorigenesis.^[^ ^54^, ^55^, ^56^ ^]^ It suggests a bacteria‐mediated anti‐CAC strategy targeting Th17‐activating pathogens.

As major innate sentinel cells that provide a first‐line defence against invading pathogens through pattern recognition receptors (PRRs), colonic DCs are pivotal in maintaining intestinal homeostasis and orchestrating innate and adaptive immune responses.^[^ ^57^ ^]^ Existing evidence supports two hypothetical mechanisms by which DCs sense gut bacteria, one is proposed to be the active sampling of bacteria by the host in an M cell‐dependent manner, and another is DCs acting as antigen‐sampling cells when bacteria have already crossed the epithelial barrier.^[^ ^58^ ^]^ Here, our study focused on the latter way that DCs contact gut bacteria, demonstrated by higher levels of serum LPS and FITC‐Dextran in *Megasphaera*‐colonized mice, together with lower expressions of tight junction proteins in the colon tissue.

To explore the direct link between *M. elsdenii* and LPDCs, we initially showed an accumulation of DCs in colonic LP, especially of the CD11b+CD103+ and CD11b+CD103‐ subsets in *M. elsdenii*‐infected mice. With phenotypic and functional analysis, a scheme of three separate CD11c^hi^ intestinal DC subsets have been distinguished: CD103+CD11b−, CD103+CD11b+ and CD103−CD11b+.^[^ ^59^, ^60^ ^]^ CD103+ DCs mainly present oral antigens and induce intestinal Treg cells differentiation contributing to immune tolerance in gut,^[^ ^61^, ^62^ ^]^ while CD11b+ DCs serve as inflammatory effector cells.^[^ ^29^ ^]^ A dramatically increased percentage of CD103− CD11b+ DCs in LP was shown in DSS treated mice colitis model, which exhibited pro‐inflammatory properties in the inflamed colon.^[^ ^62^ ^]^ CD11b+CD103+ and CD11b+CD103‐ subsets are shown to instruct Th1 and Th17 cell responses.^[^ ^30^, ^31^, ^63^ ^]^ Herein, we showed an increased number of CD11b+ DCs and higher Th1 and Th17 cell responses in *M. elsdenii*‐infected mice, suggesting the inflammatory role of this bacteria. Further research will be necessary to fully understand how the functionally distinct subsets of LPDCs orchestrate immune responses to particular pathogens or inflammatory disorders.

DCs are equipped with molecular machinery to sense foreign microorganisms and tissue injury via recognition of PAMPs and DAMPs,^[^ ^64^ ^]^ which are recognized by evolutionarily conserved, germline‐encoded PRRs that are expressed by DCs. The best‐characterized PRR family comprises the TLRs that recognize bacteria or viruses. In addition to TLRs, intracellular Nod‐like receptors and the membrane‐associated C‐type lectins function as PRRs.^[^ ^65^ ^]^ Besides activation of NF‐κB pathway, IRFs, MAPK, and cGAS‐STING pathway also involves in activation of DCs.^[^ ^65^ ^]^ With functional experiments (TLR4 antagonist, IRF4 knockdown, and purified *M. elsdenii*‐LPS pull down assay), we showed that TLR4 is the main functional mediator of *M. elsdenii*. The inflammation‐ and tumorigenesis‐promoting effect of *M. elsdenii* was negated in *Tlr4*‐deficient mice upon AOM/DSS administration, suggesting that TLR4 is crucial for the action of *M. elsdenii*. Remarkably, decreased infiltration and activation of DC were observed in *Tlr4*‐deficienct condition, which supports the hypothesis that *M. elsdenii* conferred susceptibility to CAC due to proinflammatory DCs activation. Recent reports have documented the role of IRF4 in the maturation and activation of DCs,^[^ ^36^ ^]^ controlling the development of specific DC subsets in the intestine.^[^ ^37^, ^38^ ^]^ Using in vitro IRF4 knockdown, our work suggests that *M. elsdenii* target this transcriptional factor to induce DC maturation and activation. Though we did not test additional pathways by which *M. elsdenii* activates DCs, we believe that the interaction of bacteria with TLR4 is an essential step in triggering this effect. Future structural to functional studies that identify the detailed chemical features and the epitopes responsible for immune recognition will benefit our standing of the crosstalk between *M. elsdenii* and the human host.

## 4 Conclusion

In conclusion, we have identified a novel role of *M. elsdenii* in colitis and CAC, which depends on the activation of DCs. A TLR4/NF‐κB/IRF4 signaling pathway is indispensable for *M. elsdenii*‐mediated DC activation and thereby leads to the augment of colonic Th1 and Th17 inflammation, resulting in an exacerbation of colitis‐associated tumorigenesis. Hence, this study releases a potential therapeutic strategy against colon inflammation and associated cancer by targeting of *M. elsdenii*‐enriched gut microbiota and/or its shaped mucosal immune landscape.

## 5 Experimental Section

### Meta‐Analysis of Megasphaera Across Multiple Human Cohorts

Published raw sequencing data of IBD and CRC cohorts from seven countries were separately downloaded from China national GeneBank database Nucleotide Sequence Archive (CNPhis0000042), European Nucleotide Archive database (PRJEB15371, PRJEB12449, ERP005534, SRP136711, ERA000116, ERP002061, ERP003612, ERP004605, ERP002469 and ERP002061), the Genome Sequence Archive in National Genomics Data Center China National Center (GSA‐Human: HRA005038), the NCBI Sequence Read Archive (PRJNA429990, PRJNA531273, PRJNA400072, PRJNA385949, PRJNA389927, PRJNA28117, SRA002775, SRA045646 and SRA050230) and the DNA Data Bank of Japan databases (DRA006684, DRA006684 and DRA008156). The difference in the abundance of *Megasphaera* between patients and healthy subjects for each cohort was analyzed with a nonparametric test, and *p*<0.05 was considered as statistical significance.

### Patient Recruitment and Biospecimen Collection

CRC patients were diagnosed following postoperative pathological examination of tissue biopsies collected during colonoscopy. Healthy control subjects were recruited volunteers with no gastrointestinal tumors confirmed by colonoscopy screening. Fecal samples were collected from the Fudan University Shanghai Cancer Center, Shanghai, China, and the Second Hospital of Shandong University, Shandong, China, from 2018 to 2021. Feces were obtained from patients at the hospital 2 weeks after colonoscopy and stored at −80 °C before undergoing metagenomic sequencing. Tumor stage was evaluated based on tumor size, node and metastasis (TNM) staging system. Stool samples were collected before surgery, and participants with a family history of CRC, irritable bowel syndrome, neoadjuvant therapy and other coexisting malignancies were excluded. The enrolled CRC patients were grouped as early onset (EO)‐CRC (aged <50 years) and late‐onset (LO)‐CRC (≥50 years). Ethical approval was obtained from the Institutional Review Board of Fudan University Shanghai Cancer Center (Approval No. 050432‐4‐1911D), and written informed consent was obtained from all subjects before sampling.

### Fecal Metagenomic Analysis

Raw sequencing reads were processed as we described previously,^[^ ^41^, ^66^ ^]^ quality‐filtered reads were obtained and reassembled using IDBA‐UD (V.1.1.1). The clean reads were aligned to the database (V.202003, (https://ftp.ccb.jhu.edu/pub/data/kraken2_dbs/) using Kraken2 software (V.2.1.1) and Braken software (V.2.5) to obtain species‐level information. Taxa difference among multiple groups were analyzed with the Kruskal‐Wallis test under the Benjamini, Krieger and Yekutieli method. Differential genus among healthy and CRC subjects from fecal metagenomic data was selected by *p*<0.01 and log2fc >1&←1. The charts of human fecal microbiota were plotted by an online graphic platform, Shanghai Personal Biotechnology Co.,Ltd (https://www.genescloud.cn/chart/).

### Animals

C57BL/6 mice and *Tlr4* ^−/−^ mice were purchased from GemPharmatech Co., Ltd and maintained in‐house for 1 week before any treatment. All animals (a total of 168 C57BL/6 mice and 35 *Tlr4* ^−/−^ mice) were kept in a specific pathogen‐free (SPF) environment, a 12‐h light‐dark cycle, and allowed water and standard 4% fat chow ad libitum. In each experiment, mice were age‐matched and randomly assigned to the different experimental groups. All animal studies were approved by the Animal Ethics Committee of the Shanghai University of Traditional Chinese Medicine (PZSHUTCM210715001). Germ free (GF) mice were obtained and raised at GemPharmatech Co., Ltd, where indicated were mono‐colonized with *M. elsdenii*. For DSS‐induced colitis in GF mice, 2% DSS was administered in drinking water for 7 days.

### Bacterial Culture and Administration

*M. elsdenii* (CCUG 64197T) was purchased from Culture Collection University of Gothenburg. The bacteria were cultured on EG broth medium and agar plates supplied with 5% horse blood (Topbio) under anaerobic conditions at 37 °C.

For colonization of *M. elsdenii*, bacteria were harvested and resuspended in sterile PBS to reach approximately to 1×10^9^ colony‐forming units (CFU) mL^−1^. Referring to the dose of bacteria commonly used across studies,^[^ ^67^ ^]^ mice were gavaged with 100 µl of bacterial suspension (1×10^8^ CFU per mouse) while control mice were gavaged with PBS. For mono‐colonization of *M. elsdenii* in GF mice, 1×10^9^ CFU per mouse was used. Feces were collected for quantitative PCR to detect the colonization of *M. elsdenii*.

### Antibiotic Treatment

Mice were given the following antibiotic cocktail (ABX) via oral gavage (200 µL per mouse) for 2 weeks: ampicillin (1 g L^−1^, Aladdin), neomycin (1 g L^−1^, Aladdin), vancomycin (500 mg L^−1^, Aladdin), and metronidazole (1 g L^−1^, Aladdin). Feces were collected for quantitative PCR to detect colon sterilization.

### AOM/DSS Murine Colitis‐Associated Cancer (CAC) Model

Following the method of modeling AOM/DSS in the previous study,^[^ ^68^ ^]^ mice were intraperitoneally injected with 12.5 mg kg^−1^ body weight of AOM (MP Biomedicals), and after 1 week, mice were subjected to three cycles of 2.5% DSS (MP Biomedicals). Each cycle lasted 7 days and was allowed for a 2‐week recovery period. On the day of DSS treatment, they were gavaged by 1×10^8^ CFU of *M. elsdenii* or PBS. The disease activity index (DAI) scoring system was determined by the body weight change, occult or gross blood presence, and stool consistency (Table S2, Supporting Information). Mice were sacrificed at the indicated time for tumor and tissue analysis. Standard transverse or longitudinal sections were used for histological staining (H&E, Alcian blue staining for goblet cells, and immunohistochemistry) to observe colonic inflammation and tumor pathology. The inflammation score was assigned referring to Barthel et al. (Table S3, Supporting Information)^[^ ^69^ ^]^ and the tumor pathology was evaluated as previously described.^[^ ^70^, ^71^ ^]^

### Intestinal Permeability Assay

Mice were orally administrated with 4 kDa FITC‐dextran (Sigma‐Aldrich, 0.4 mg g^−1^ body weight) 4 h before blood collection. Serum FITC‐dextran was carried out using a multimode microplate reader (Spark, Tecan) at the excitation of 485 nm and emission of 535 nm. Dilution of FITC‐dextran at known concentrations was used as standard curves while serum from PBS‐gaveged mice was used as background control.

### LPS Measurement

Colonic tissues were harvested and homogenized in PBS containing protease inhibitor (Beyotime) and centrifuged to obtain supernatant. Then, serum LPS and colonic tissue LPS concentration were quantified using a mouse LPS ELISA kit (Coibo Bio) according to the manufacturer's protocols. The optical density was measured at the absorption of 450 nm using the multimode microplate reader (Spark, Tecan).

### LPS Purification and Biotin labeling

LPS from was extracted from *M. elsdenii* using a Bacterial LPS Extraction Kit (Solarbio). LPS‐derived from *M. elsdenii* was coupled to biotin using Biotin Quick Labeling Kit with Biotin‐LC‐NHS (Beyotime) following the manufacturer's instructions. LPS‐derived from *E. coli* (Sigma) was also coupled to Biotin.

### Agarose Resin‐Based Pull‐Down Assay

Streptavidin Agarose Resin was incubated with biotin‐labelled LPS, in the presence of BMDC cell lysates using Biotinylated Protein Pull‐down Kit (Beyotime) according to the manufacturer's instructions. Target protein expressing were detected followed by SDS‐PAGE and immunoblotting.

### Quantitative Real‐Time PCR

Fecal DNA was extracted using the QIAamp Fast DNA Stool Mini Kit (Qiagen) in accordance with the manufacturer's instructions. Total RNA from colonic tissues or cultured cells was isolated using Trizol reagent (Invitrogen), and cDNA was subsequently synthesized using the Hifair AdvanceFast 1st Strand cDNA Synthesis Kit (Yeason). Quantitative real‐time PCR (q‐PCR) was performed using Hieff qPCR SYBR Green Master Mix (Yeason). Primers of target genes were listed in Table S1 (Supporting Information).

### Chromatin Immunoprecipitation (ChIP)‐qPCR Assay

ChIP assay was performed using ChIP Kit (Bioruqi) according to the manufacturer's protocol. Immunoprecipitation was performed with Anti‐IRF4 antibody (CST) or rabbit IgG (Abclonal). DNA was purified for qPCR analyses using Hieff qPCR SYBR Green Master Mix (Yeason). Primers of target genes used for ChIP qRT‐PCR were listed in Table S1 (Supporting Information).

### Cell Processing

Colonic lamina propria (LP) cells were isolated as previously described with modifications.^[^ ^72^ ^]^ Briefly, the colon was removed from mesenteric fat tissue and Peyer's patches (PPs), opened longitudinally, cut into 1 cm pieces, and washed in cold PBS. After incubation with EDTA (Beyotime) and dithiothreitol (Beyotime) in Hanks’ balanced salt solution for 20 min, tissue homogenates were filtered through a 70 mm cell strainer. The remaining LP with muscle layer was collected and subsequently incubated with a digestion solution containing collagenase (Sigma‐Aldrich), DNase I (Roch) and dispase (Beyotime) for 40 min and then filtered through a 70 mm cell strainer. Collected suspensions were centrifuged for 10 min at 1200 rpm, washed in ice‐cold PBS and resuspended in FACS buffer (BD Biosciences). The single‐cell suspensions were then used for further flow cytometric analysis.

To obtain BMDC, BM was flushed with RPMI medium and passed through a 70 mm cell strainer to yield a single cell suspension. Then cells were treated with RBC lysis buffer to remove red blood cells. Cells were cultured in RPMI complete medium supplemented with 20 ng mL^−1^ recombinant murine GM‐CSF (Peprotech) and 10 ng mL^−1^ IL4 (Peprotech), and the medium were half‐exchanged every second day. BM‐derived cells were collected on day 6, subculture at 24‐well plate, and stimulated with LPS (100 ng mL^−1^, Sigma), or co‐cultured with the indicated ratio (MOI 100:1) of *M. elsdenii* for another 24 h and afterwards harvested for morphological analysis with Giemsa staining (Beyotime), RNA‐seq, or flow cytometry. In indicated experiments, *M. elsdenii*‐infected BMDCs were treated with selected TLR2 antagonist (TLR2‐IN‐C29, Selleck) or TLR4 antagonist (MD2‐IN‐1, Selleck) at a concentration of 10 × 10^−6^ m.

To isolate spleen CD4+ T cells, spleen was extracted and mechanically minced and pressed using syringe plungers through a 70 mm cell strainer. After removing the red blood cells (RBC) with RBC lysis buffer, the CD4+ T cells were enriched by negative selection using CD4+ T Cell Isolation Kit (Miltenyi) as described by the manufacturer's protocols. To expand and activate CD4+ T cells, cells were incubated with plate‐coated CD3 antibody (BD Biosciences, 1 mg mL^−1^) and soluble CD28 antibody (BD Biosciences, 2 mg mL^−1^L) along with 50U recombinant murine IL2 (Peprotech) for 6 days. Where indicated, CD4+ T cells were treated with *M. elsdenii* for another 72 h. To establish co‐culture system of BMDCs and spleen CD4+ T cells, BMDC were pretreated with *M. elsdenii* for 24 h and then cocultured with CD4+ T cells at a ratio of 1:10 for 72 h. CD4+ T cells were then harvested for qPCR or flow cytometric analysis.

### RNA‐Seq Analysis of BMDCs

BMDCs were infected with *M. elsdenii* for 24 h as described above; then total RNA was isolated and qualified by agarose gel electrophoresis. The RNA concentration and the ratio of 260/280 were determined by Nanodrop One Microvolume UV‐Vis Spectrophotometer (Thermo Fisher Scientific). cDNA libraries were constructed and sequenced by Majorbio Biotech. The data analysis was performed on Majorbio cloud platform (https://cloud.majorbio.com/). For Gene Set Enrichment Analysis (GSEA), predefined gene sets from the Molecular Signatures Database (MSigDB v5.0) was used. In the present study, the immune signatures were focused, C7 collection was used (highly conserved between humans and mouse models) for GSEA analysis.^[^ ^73^ ^]^ List of ranked genes was based on Signal2Noise and the minimum and maximum criteria for selection of gene sets from the C7 collection were 15 and 500 genes respectively. The accession number for the sequencing data is GSE279592.

### Flow Cytometry

Single‐cell suspensions were incubated with TruStain FcX (Biolegend) to block Fc receptors and then stained with surface markers for 30 min at room temperature. For intracellular cytokine analysis, cultured cells were stimulated with 1× Cell Stimulation Cocktail plus protein transport inhibitors (eBioscience) for 6 h. Cells were fixed and permeabilized with Cytofix/Cytoperm Fixation/Permeabilization Kit (BD Biosciences) and stained with indicated intracellular antibodies for 50 min at room temperature. Antibodies were obtained from Biolegend and listed in Table S1 (Supporting Information). Samples were then detected using CytoFlex Flow Cytometer (Beckman), and data were analyzed with FlowJo software (Treestar).

### Cell Sorting

To obtain different colonic DC subsets, LPMCs were sorted on CytoFLEX SRT (Beckman Coulter). The obtained CD103+ CD11b‐ DC subset and CD11b+ DC subset were then co‐cultured with spleen CD4+ T cells at a ratio of 1:10 in the presence of *M. elsdenii* or not for 72 h. CD4+ T cells were then harvested for qPCR.

### Plasmids Transfection

IRF4 and control shRNA plasmids were generated by Genechem. BMDCs were sub‐cultured at 6‐well plate for another 24 h, and then transfected with IRF4 shRNA plasmids or vector, after transfection for 48 h, BMDCs were co‐cultured with the indicated ratio (MOI 100:1) of *M. elsdenii* for another 24 h. Plasmid transfections were conducted using Lipofectamine 3000 (Invitrogen), according to the manufacturer's instructions.

### H&E Staining, Alcian‐Blue Staining and Immunohistochemistry

Paraffin‐embedded 4‐µm sections were dewaxed and rehydrated in a standardized procedure. Hematoxylin and eosin staining were performed by H&E Staining Kit (Beyotime). Alcian Blue staining was performed using the Alcian Blue & Nuclear Fast Red Staining Kit (Beyotime). For immunohistochemistry (IHC), after being dewaxed, rehydrated, and treated with hydrogen peroxidase, sections were processed for antigen retrieval in citrate buffer using microwaves. Then the sections were incubated with a primary rabbit anti‐mouse polyclonal Zo‐1 antibody (Proteintech) and with a primary rabbit anti‐mouse monoclonal Occludin antibody (Zenbio) at the appropriate dilution for 16 h at 4 °C. The visualization of positive signals was performed using UltraSensitive SP IHC Kit (Maxim) according to the manufacturer's instructions. The mean densities of Zo‐1 and Occludin in the colon were calculated by Image J with 5 random microscopic fields for each sample.

### Western Blotting

The intracellular protein extraction, SDS‐PAGE, protein transfer, antibody incubation and immunoblotting detection were performed as previously described.^[^ ^74^ ^]^ The antibodies used for western blotting were listed in the Table S1 (Supporting Information).

### Fecal Microbiota Transplantation (FMT)

For FMT experiments, fecal samples were collected from healthy donors, and CRC patients were grouped according to the expression level of *M. elsdenii* (n = 3 per group). Bacteria‐enriched supernatants were collected by centrifugation and resuspended in sterilized saline, and stored at ‐80 °C. After receiving an ABX for 4 weeks, recipient mice surrendered AOM/DSS cycles and orally gavaged with fecal microbiota transplants from each donor group on the day of DSS treatment 3 times per week until sacrificed.

### Statistical Analysis

Statistical analysis was performed using GraphPad Prism 10.0.0 (GraphPad Software). All data presented as mean ± SEM. As stated, statistical significance was determined by a two‐tailed Student's t‐test to compare the differences between two groups, one‐way ANOVA with the Tukey method or two‐way ANOVA for multiple‐group comparisons. Differences with *p* values< 0.05 were considered significant. *, *p*< 0.05; **, *p*< 0.01; ***, *p*< 0.001, ****; *p*< 0.0001. All statistical details regarding p‐value and n can be found in the figure legends and supplementary figure legends.

## Conflict of Interest

The authors declare no conflict of interest.

## Author Contributions

X.H., H. G., Y. M., and L. Z. conceived and designed the study. X. H. and Z. Z. performed in vivo, *ex vivo*, and in vitro studies, analyzed data and wrote the paper. W. C., X. Z., M. L. and X. G. developed mouse models and analyzed data. J. L. and Y. M. provided human CRC feces and metagenomic data analysis. H. G., Y. M. and L. Z. reviewed the manuscript. All authors approved the final version. X. H., Z. Z., Q. C., J. L. contributed equally to this work.

## Supporting information



Supporting Information

## Data Availability

The data that support the findings of this study are openly available in Genome Sequence Archive in National Genomics Data Center China National Center at https://ngdc.cncb.ac.cn/gsa‐human/, reference number 32.
