# Supplementary material for: Megasphaera Elesdenii Dysregulates Colon Epithelial Homeostasis, Aggravates Colitis‐Associated Tumorigenesis
Source: Adv Sci (Weinh). 2025 Aug 13;12(41):e05670. doi: 10.1002/advs.202505670 (PMC12591162; doi:10.1002/advs.202505670)
Supplement: Supplementary file 1 — Supporting Information [file ADVS-12-e05670-s001.docx]

***Megasphaera Elsdenii* Dysregulates Colon Epithelial Homeostasis, Aggravates Colitis-associated Tumorigenesis**

Xinxin Hou, Zhaozhou Zhang, Wanqing Chen, Jinmin Li, Xiangxiang Zhu, Mingjie Li, Xiaoqi Guan, Haidong Guo*, Yanlei Ma* and Ling Zhao*

**Supplementary Figure**


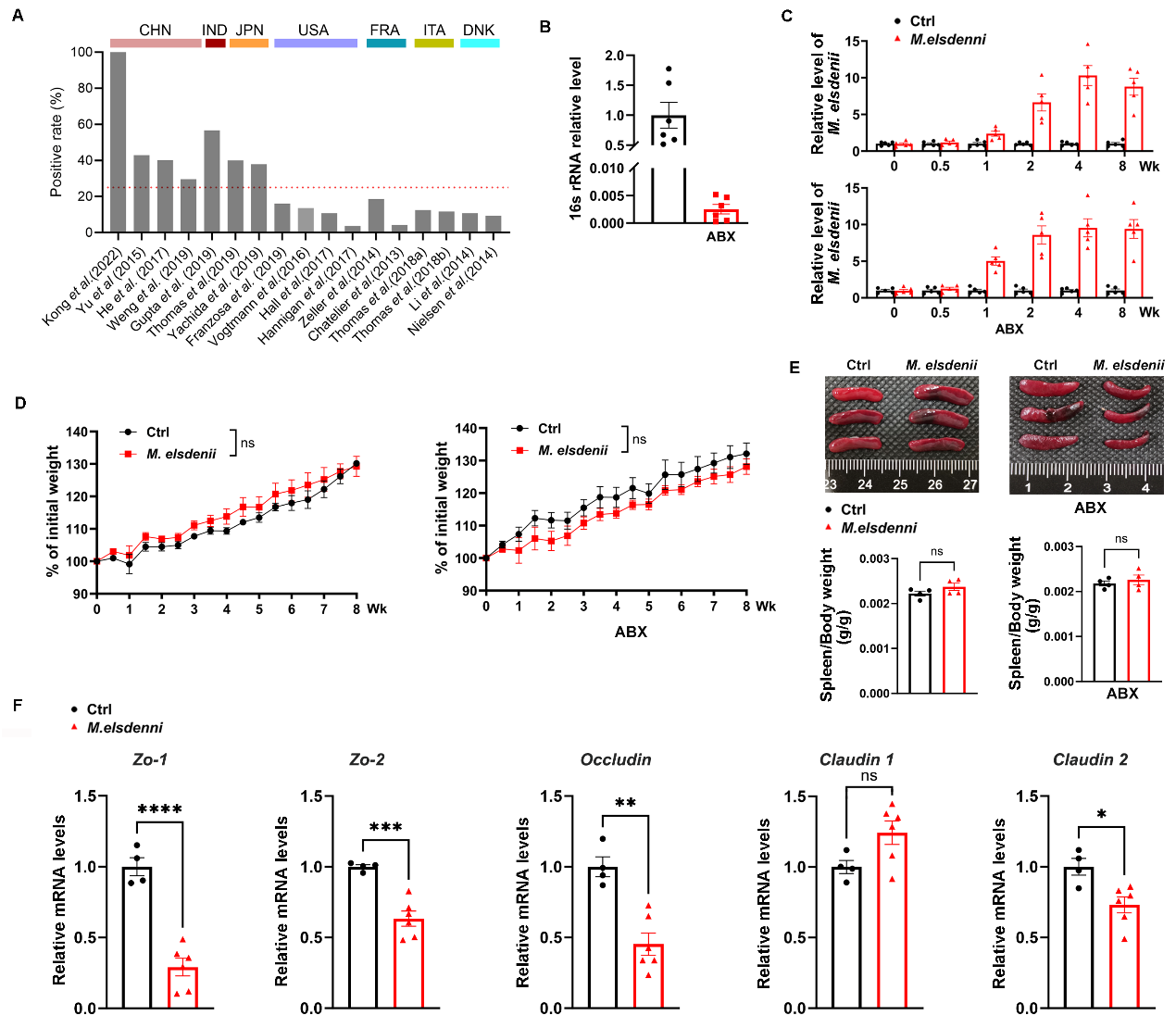


**Figure S1.** ***M. elsdenii* Shows no Systemic Harm to the Host, Related to Figure 1.** (A) The positive rate of genus *Megasphaera* across cohorts. Red dashed line denotes positive rate of 25%. (B, C) The level of total bacteria on the final day of ABX treatment (B) and the level of *M. elsdenii* during *M. elsdenii* gavage period (C) in stool samples was determined by qPCR (n=5-6/group). (D) The change in the body weight of SPF (left) or ABX-treated mice (right) during *M. elsdenii* gavage period (n=7/group). (E) Representative pictures of the spleen and spleen index from SPF (left) or ABX-treated mice sacrificed after *M. elsdenii* gavage period. (F) The mRNA levels of tight junction proteins (*Zo-1*, *Zo-2*, *Occludin*, *Claudin1* and *Claudin2*) resulted from qPCR (n=4 in control group, n=6 in *M. elsdenii* group). Each experiment conducted at least 2-3 replicates. Data presented as mean ± SEM. Statistical analysis was performed with two-way analysis of variance (ANOVA) in (D), and Student’s t test in (E, F); ns, not significant, **p* < 0.05, ***p* < 0.01, ****p* < 0.001, *****p*<0.0001.

**
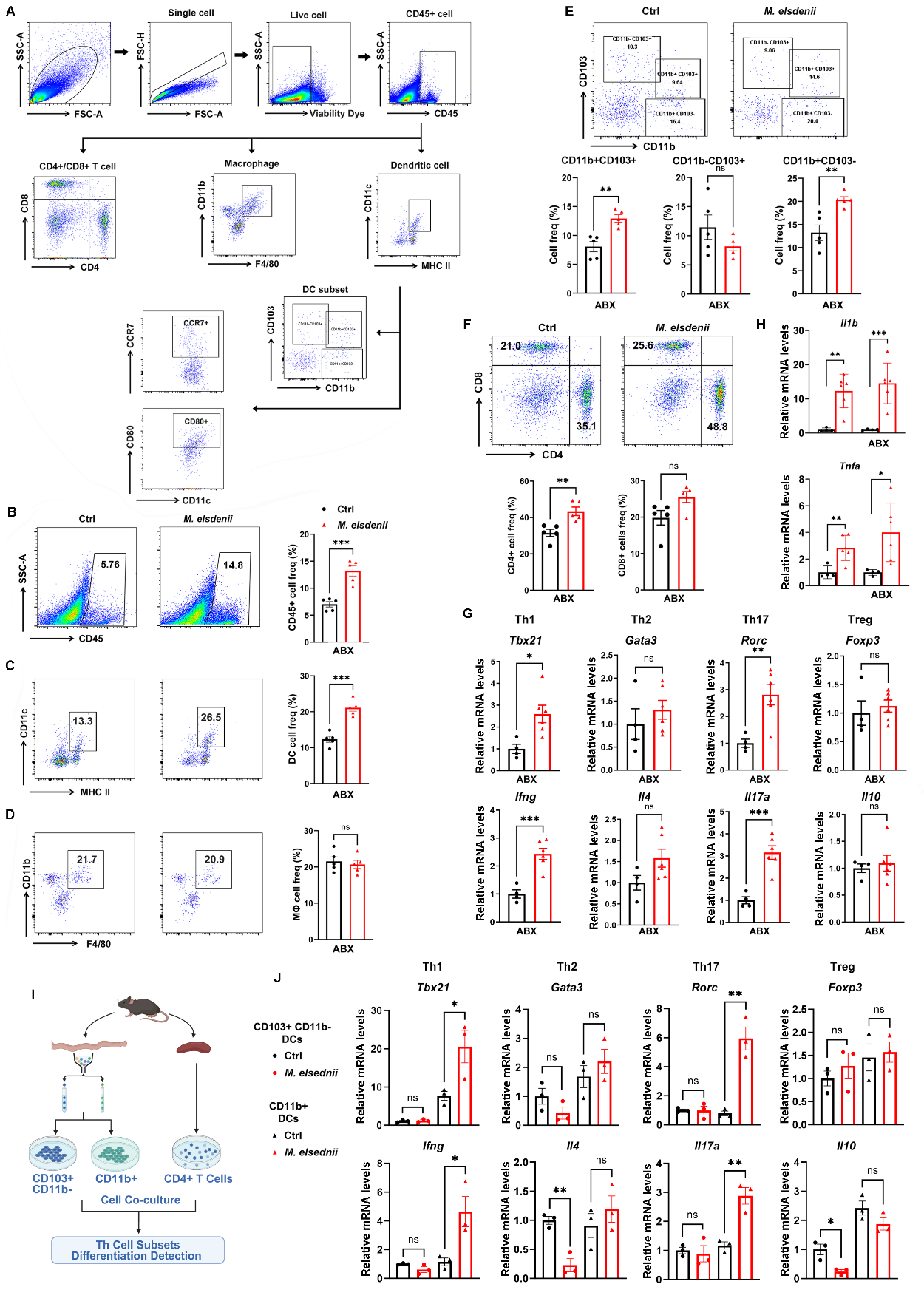
**

**Figure S2.** **Colonization of *M. elsdenii* remodels the colonic mucosal immune landscape, Related to Figure 2.** (A) Gating strategy of colonic lamina propria (LP) immune cells amongst colon digests. (B) Flow cytometric analysis of CD45+ hematopoietic cells after gavaged with *M. elsdenii* (or not) for eight weeks in ABX-treated mice (n=5/group). (C, D) Flow cytometric analysis of CD11c+ MHC II+ dentritic cells (C) and CD11b+ F4/80+ macrophages (D) in the colonic LP of *M. elsdenii*-colonized (or not) ABX-treated mice (n=5/group). (E) Flow cytometric analysis of DC cell subsets shows the abundance of CD11b+ CD103+ DC, CD11b- CD103+ DC and CD11b+ CD103- DC (n=5/group) in ABX-treated mice. (F) Flow cytometric analysis of CD4+ and CD8+ T cells in the colonic LP of *M. elsdenii*-colonized (or not) ABX-treated mice (n=5/group). (G) qPCR analysis of Th1-, Th2-, Th17-, and Treg-associated transcription factor (*Tbx21*, *Gata3*, *Rorc* and *Foxp3*) and cytokine (*Ifng*, *Il4*, *Il17a* and *Il10*) in the colonic tissue of *M. elsdenii*-colonized (or not) ABX mice (n=4 in control group, n=6 in *M. elsdenii* group). (H) qPCR analysis of proinflammatory cytokines *Il1b* and *Tnfa* in the colon tissue of *M. elsdenii*-colonized (or not) mice (n=4 in control group, n=6 in *M. elsdenii* group). (I) Schematics of *ex vivo* colonic DC subsets sorting and spleen CD4+ T cell co-culture system. (J) qPCR analysis of Th1-, Th2-, Th17-, and Treg-associated transcription factors (*Tbx21*, *Gata3*, *Rorc* and *Foxp3*) and cytokines (*Ifng*, *Il4*, *Il17a* and *Il10*) in CD4+ T cells in the DC subset co-culture system (n=3 independent experiments). Each experiment conducted at least 2-3 replicates. Data presented as mean ± SEM. Statistical analysis was performed with Student’s t test; ns, not significant, **p* < 0.05, ***p* < 0.01, ****p* < 0.001.


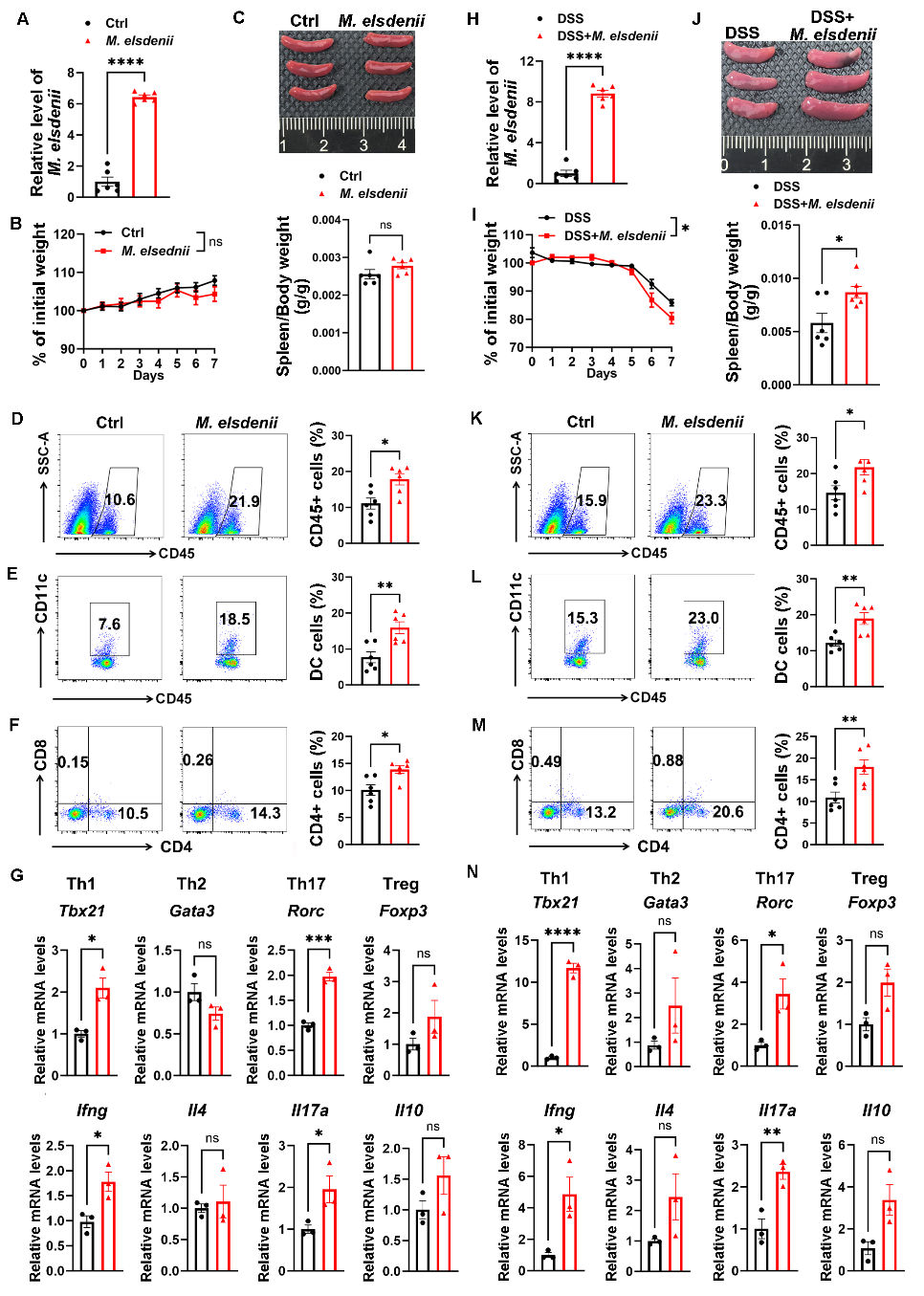


**Figure S3 Colonization of *M. elsdenii* in germ free mice exerts colonic inflammation and induces more severe DSS-induced colits, Related to Figure 3.** (A) Level of *M. elsdenii* during *M. elsdenii* gavage period in stool samples of GF mice was determined by qPCR (n=6/group). (B) The change in the body weight of mice during *M. elsdenii* gavage period (n=6/group). (C) Representative pictures of the spleen and spleen index after *M. elsdenii* gavage period. (D) Flow cytometric analysis of colonic LP CD45+ hematopoietic cells after gavaged with *M. elsdenii* (n=6/group). (E) Flow cytometric analysis of CD11c+ DCs in the colonic LP (n=6/group). (F) Flow cytometric analysis of CD4+ and CD8+ T cells in the colonic LP (n=6/group). (G) qPCR analysis of Th1-, Th2-, Th17-, and Treg-associated transcription factors (*Tbx21*, *Gata3*, *Rorc* and *Foxp3*) and cytokines (*Ifng*, *Il4*, *Il17a* and *Il10*) in colonic tissues (n=3/group). (H) Level of *M. elsdenii* during *M. elsdenii* gavage period in stool samples of GF DSS mice was determined by qPCR (n=6/group). (I) The change in the body weight of GF DSS mice during *M. elsdenii* gavage period (n=6/group). (J) Representative pictures of the spleen and spleen index after *M. elsdenii* gavage period in GF DSS mice. (K) Flow cytometric analysis of colonic LP CD45+ hematopoietic cells after gavaged with *M. elsdenii* in GF DSS mice (n=6/group). (L) Flow cytometric analysis of CD11c+ DCs in the colonic LP of GF DSS mice (n=6/group). (M) Flow cytometric analysis of CD4+ and CD8+ T cells in the colonic LP of GF DSS mice (n=6/group). (N) qPCR analysis of Th1-, Th2-, Th17-, and Treg-associated transcription factors (*Tbx21*, *Gata3*, *Rorc* and *Foxp3*) and cytokines (*Ifng*, *Il4*, *Il17a* and *Il10*) in colonic tissues of GF DSS mice (n=3/group). Each experiment conducted at least 2-3 replicates. Data presented as mean ± SEM. Statistical analysis was performed with Student’s t-test and two-way ANOVA in (B, I); ns, not significant, **p* < 0.05, ***p* < 0.01, ****p* < 0.001.

**
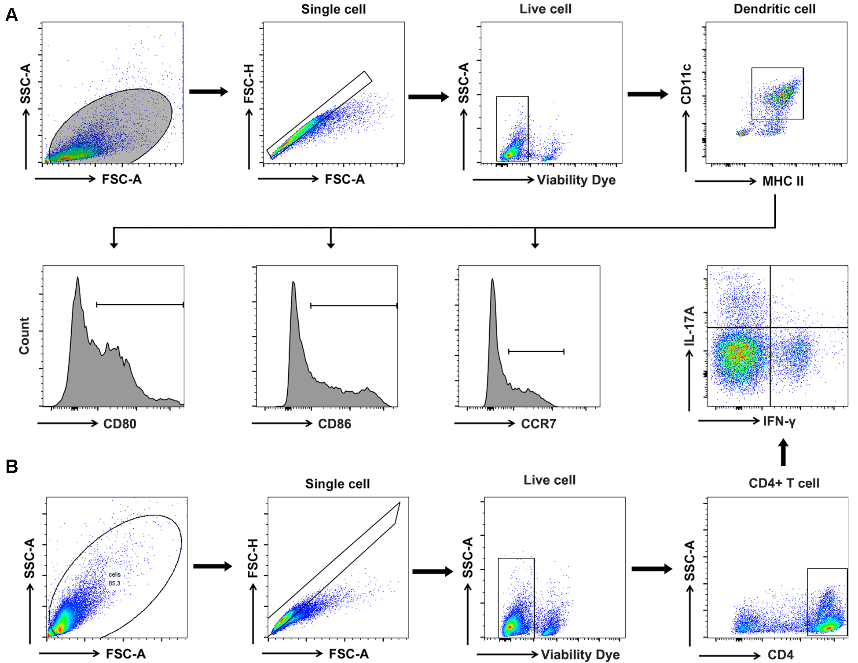
**

**Figure S4. *M. elsdenii* induces Th1 and Th17 immune response through DC cell activation, Related to Figure 4.** (A) Gating strategy of CD80, CD86 and CCR7 expression in BMDCs treated with *M. elsdenii*. (B) Gating strategy of CD4+IFNγ+ and CD4+IL17A+ T cells in spleen-isolated CD4+ T cells treated with *M. elsdenii*.

**
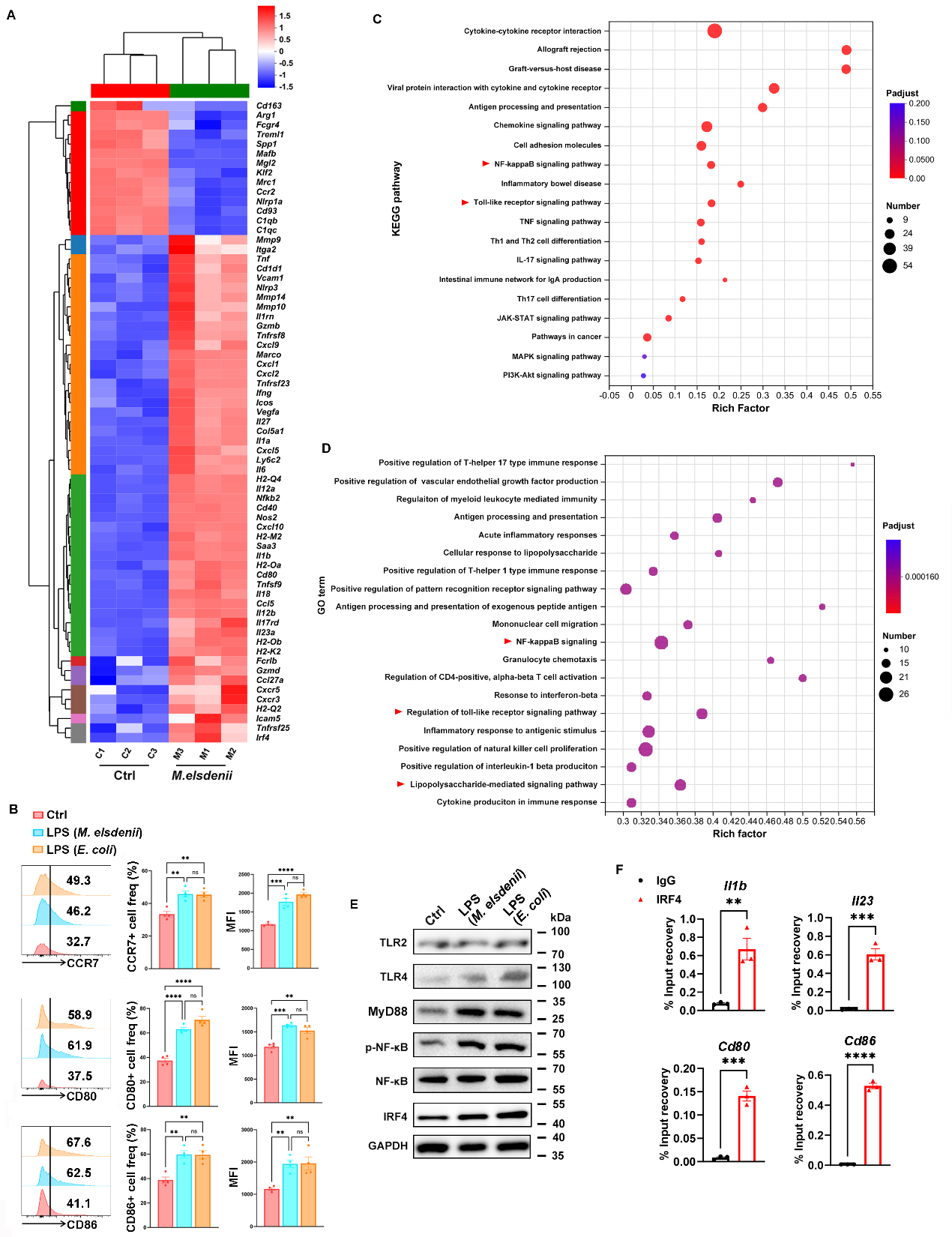
**

**Figure S5. *M. elsdenii* activates DCs through TLR4/NF-κB/IRF4 pathway, Related to Figure 4.** (A) Heatmap of selected DEGs from RNA sequencing dataset derived from biological triplicates. (B) Flow cytometric analysis of CCR7, CD80 and CD86 expression in *M. elsdenii*-drived LPS or *E. coli*-derived LPS treated BMDCs (n=4 independent experiments). (C, D) KEGG pathway enrichment (C) or GO term enrichment analysis (D) of DEGs from RNA sequencing dataset. (E) Western blot analysis of the TLR4/NF-κB/IRF4 signaling pathway in *M. elsdenii*-drived LPS or *E. coli*-derived LPS treated BMDCs. (F) Enrichment of *Il1b*, *Il23*, *CD80*, *CD86* promoter region binding of IRF4 was determined by ChIP-qRCR analysis (n=3 independent experiments). Each experiment conducted at least three replicates. Data presented as mean ± SEM. Statistical analysis was performed with one-way ANOVA (B) and Student’s t-test (F); ns, not significant, **p* < 0.05, ****p* < 0.001, *****p* < 0.0001.

**
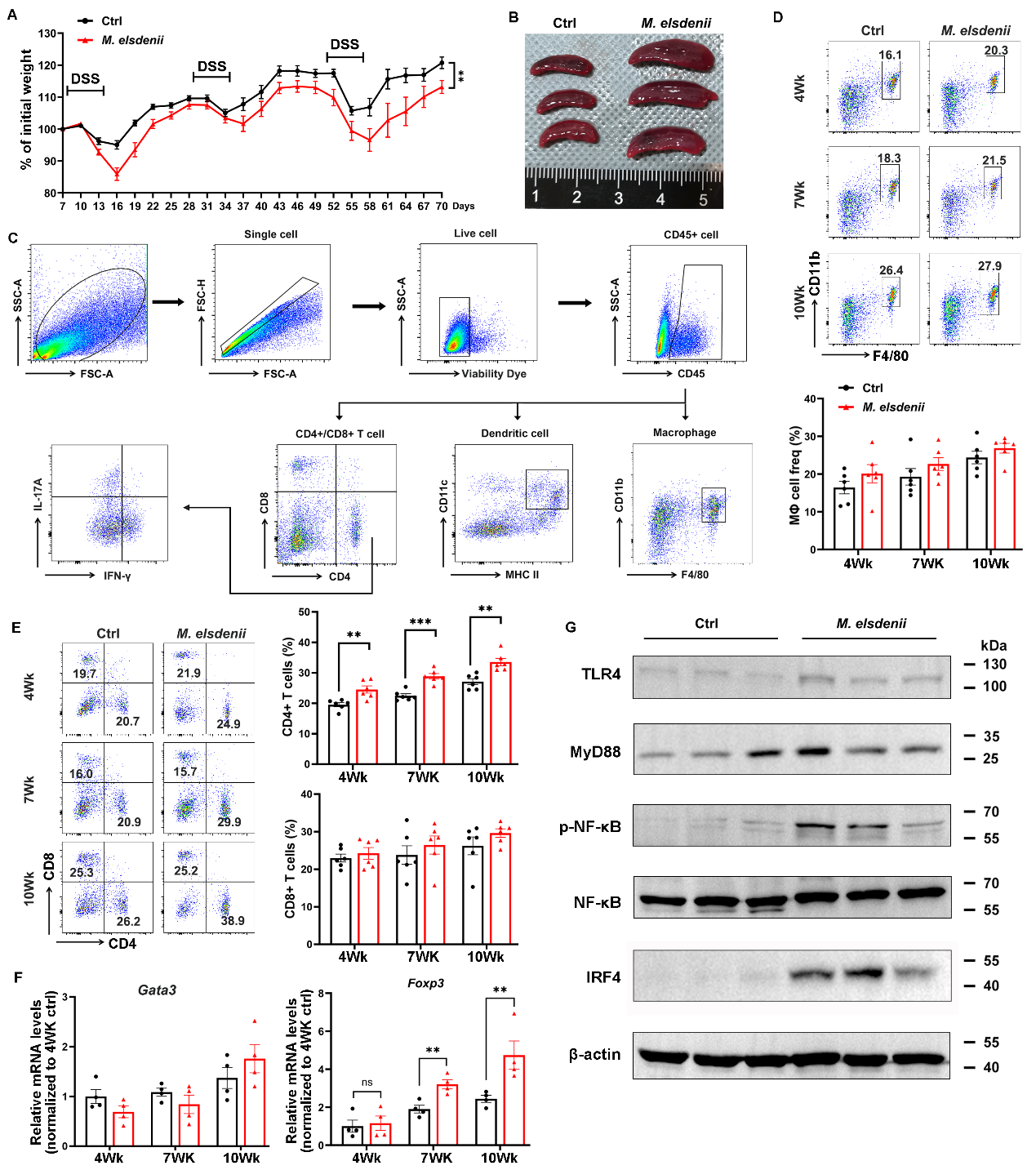
**

**Figure S6. *M. elsdenii* colonization exacerbates colonic inflammation and promotes colitis-associated tumor formation, Related to Figure 6.** (A) The change in the body weight of *M. elsdenii*-treated (or not) AOM/DSS mice (week4/7/10: n=6-8/group). (B) Representative pictures of the spleen from *M. elsdenii*-treated (or not) AOM/DSS mice sacrificed at week10 endpoint. (C) Gating strategy of LP immune cells amongst colon digests. (D, E) Flow cytometric analysis of CD11b+ F4/80+ macrophage (D), CD4/CD8+ T cells (E) and their frequencies in the colonic LP of *M. elsdenii*-treated (or not) AOM/DSS mice (week4/7/10: n=6/group). (F) qPCR analysis of Th2-related *Gata3* and Treg-related *Foxp3* in the colon tissue of *M. elsdenii*-colonized (or not) AOM/DSS mice (week4/7/10: n=4/group). (G) Western blot analysis of the TLR4/NF-κB/IRF4 signaling pathway in the colonic tissues of *M. elsdenii*-treated mice. Each experiment conducted at least 2-3 replicates. Data presented as means ± SEM. Statistical analysis was performed with Student’s t test (D-F), two-way ANOVA (A); ***p* < 0.01.

**
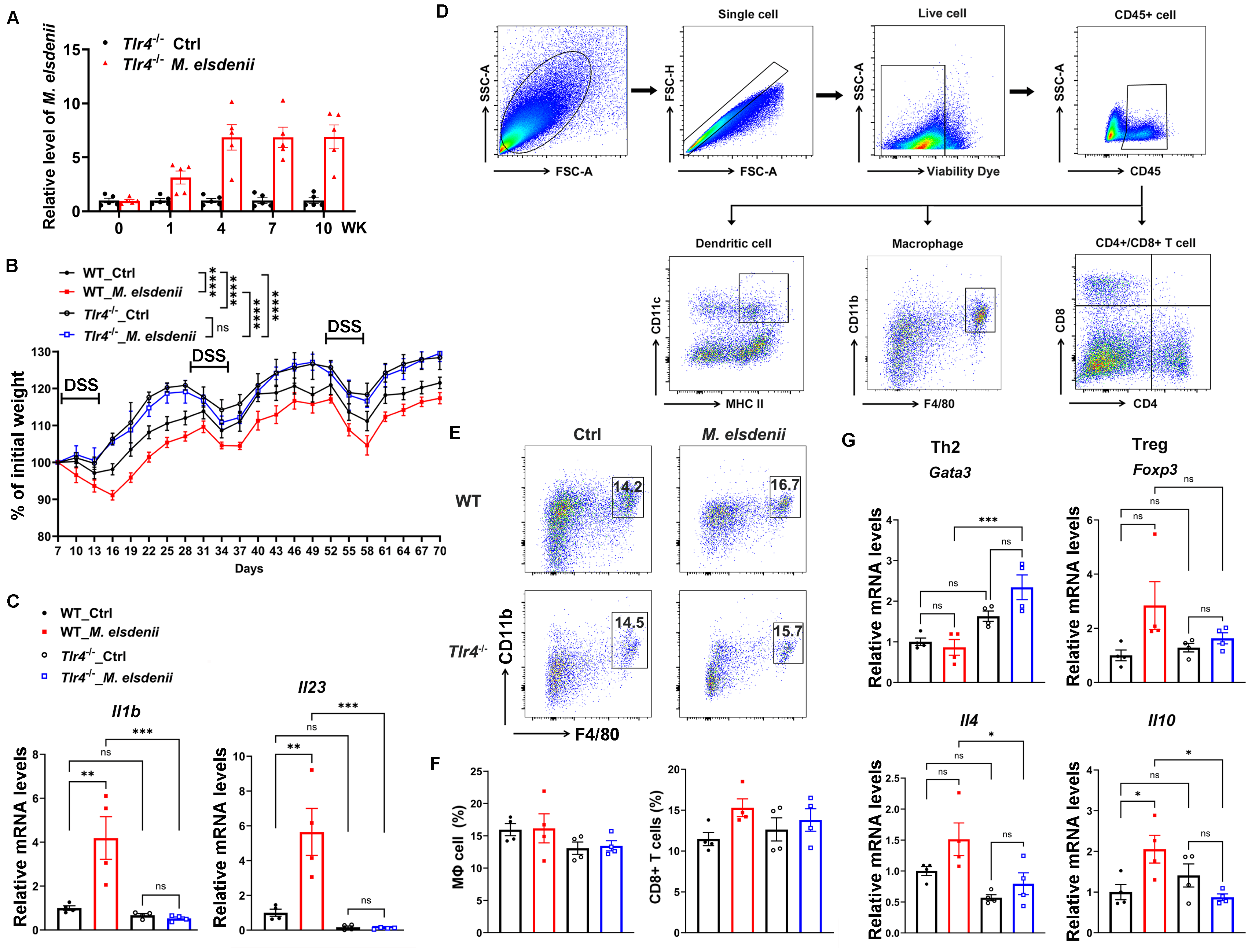
**

**Figure S7.** ***Tlr4*-deficiency prevents CAC tumorigenesis and inflammation upon *M. elsdenii* colonization, Related to Figure 7.** (A) Level of *M. elsdenii* in stool samples of *Tlr4*^-/-^ mice was determined by qPCR (n=5/group). (B) Changes in the body weight of *M. elsdenii*-colonized WT and *Tlr4*^-/-^ AOM/DSS mice (n=7-8/group). (C) qPCR analysis of DC-activated proinflammatory genes *Il1b* and *Il23* in the colon tissue of *M. elsdenii*-colonized WT and *Tlr4*^-/-^ AOM/DSS mice (n=4/group). (D) Gating strategy of LP immune cells amongst colon digests. (E, F) Flow cytometric analysis of CD11b+ F4/80+ macrophage and CD8+ T cells frequency in colonic LP of *M. elsdenii*-colonized (or not) WT and *Tlr4*^-/-^ AOM/DSS mice (n=4/group). (G) qPCR analysis of Th2- and Treg-associated transcription factors (*Gata3* and *Foxp3*) and cytokines (*Il4* and *Il10*) in the colon tissue (n=4/group). Each experiment conducted at least 2-3 replicates. Data presented as mean ± SEM. Statistical analysis was performed with one-way ANOVA (C, F, G) and two-way ANOVA (B); **p* < 0.05, ***p* < 0.01, ****p* < 0.001, *****p* < 0.0001.

**
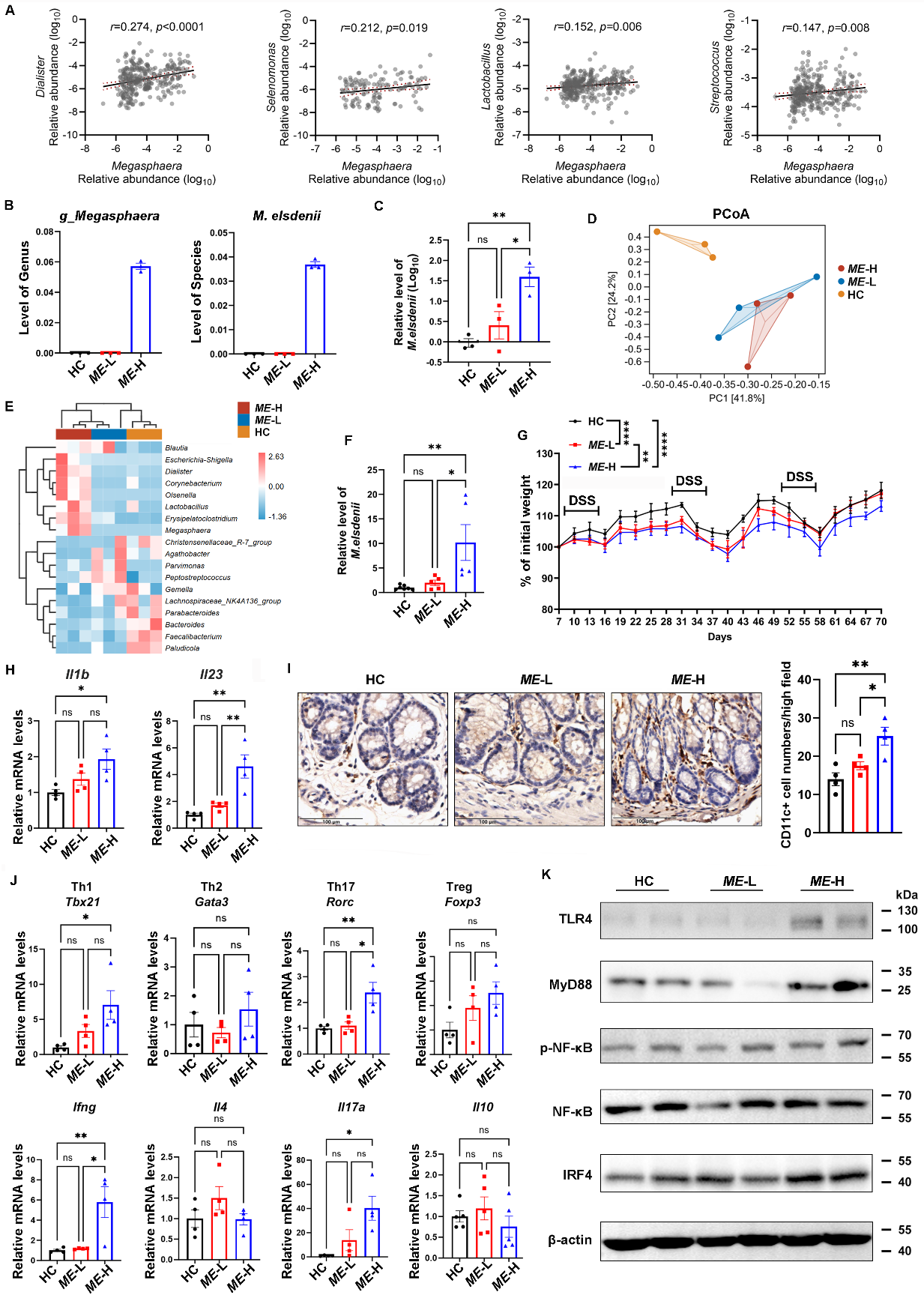
**

**Figure S8. Transplanting of *M. elsdenii*-abundant fecal microbiota from CRC patients accelerates colitis-associated tumorigenesis, Related to Figure 8.** (A) Nonparametric Spearman correlation of the abundance *Megasphaera* and *Dialister*, *Selenomonas*, *Lactobacillus* and *Streptococcus*. (B) Fecal metagenomic sequencing showing genus *Megasphaera* (left) and species *M. elsdenii* (right) abundance (n=3/group). (C) qPCR analysis of *M. elsdenii* in the feces of donors (n=3/group). (D) OUT-based PCoA analysis of donors’ microbiota (n=3/group). (E) Heatmap of differential genera among donors’ microbiota (n=3/group). (F) qPCR analysis of *M. elsdenii* in the feces of recipient mice at endpoint (n=5/group). (G) The change in the body weight of recipient mice (n=7-8/group). (H) qPCR analysis of DC-activated proinflammatory genes *Il1b* and *Il23* in the colon tissue of recipient mice (n=4/group). (I) Representative images of CD11c IHC staining and analysis of CD11c+ cell numbers in the colonic tissues in HC, *ME*-L and *ME*-H groups, scale bar=100μm. (J) qPCR analysis of Th1-, Th2-, Th17- and Treg-associated transcription factors (*Tbx21*, *Gata3*, *Rorc* and *Foxp3*) and cytokines (*Ifng*, *Il4*, *Il17a* and *Il10*) in the colonic tissues of recipient mice (n=4/group). (K) Western blot analysis of the TLR4/NF-κB/IRF4 signaling pathway in the colonic tissues of *ME*-H group relative to the HC and *ME*-L groups. Each experiment conducted at least 2-3 replicates. Data presented as mean ± SEM. Statistical analysis was performed with one-way ANOVA (B, E, G, H, I), and two-way ANOVA (F); ns, not significant, **p* < 0.05, ***p* < 0.01.

**Table S1.** **Key resources table.**

| REAGENT or RESOURCE | SOURCE | | IDENTIFIER |
| --- | --- | --- | --- |
| Antibodies |  | |  |
| FITC-Anti-mouse CD45 | Biolegend | | Cat# 147710 |
| PerCP/Cy5.5- Anti-mouse CD45 | Biolegend | | Cat# 103130 |
| FITC- Anti-mouse CD11b | Biolegend | | Cat#101206 |
| PerCP/Cy5.5-Anti-mouse/human CD11b | Biolegend | | Cat# 101228 |
| PE/Cy7-Anti-mouse F4/80 | Biolegend | | Cat# 123114 |
| APC/Cy7-Anti-mouse CD11c | Biolegend | | Cat# 117324 |
| APC-Anti-mouse I-A/I-E (MHCII) | Biolegend | | Cat# 107614 |
| APC-Anti-mouse CD103  PE**-**Anti-mouse CD4 | Biolegend  Biolegend | | Cat# 121414  Cat# 100408 |
| APC-Anti-mouse CD8 | Biolegend | | Cat# 126614 |
| PerCP/Cy5.5-Anti-mouse CD80 | Biolegend | | Cat# 104722 |
| FITC-Anti-mouse CD86 | Biolegend | | Cat# 159220 |
| PE/Cy7-Anti-mouse CCR7 | Biolegend | | Cat# 120124 |
| APC-Anti-mouse IFN-γ | Biolegend | | Cat# 505810 |
| Brilliant Violet 510 Anti-mouse IFN-γ | Biolegend | | Cat# 505841 |
| APC/Cy7-Anti-mouse IL17A | Biolegend | | Cat# 506940 |
| Zombie Violet™ Fixable Viability | Biolegend | | Cat# 423113 |
| Rabbit anti-mouse TLR2 | Cell Signaling | | Cat# 13744 |
| Rabbit anti-mouse TLR4 | Cell Signaling | | Cat# 14358 |
| Rabbit anti-mouse MyD88 | Cell Signaling | | Cat# 4283 |
| Rabbit anti-mouse IRF4 | Cell Signaling | | Cat# 15106 |
| Rabbit anti-mouse NF-κB p65 | Cell Signaling | | Cat# 8242 |
| Rabbit anti-mouse Phospho-NF-κB p65 | Cell Signaling | | Cat# 3033 |
| Hamster anti-mouse CD3e | BD Biosciences | | Cat# 567115 |
| Hamster anti-mouse CD28 | BD Biosciences | | Cat# 553295 |
| Rabbit anti-mouse Zo-1 | Proteintech | | Cat# 21773-1-AP |
| Rabbit anti-mouse Occludin | Zenbio | | Cat# R381549 |
| Anti-RNA polymerase II RPB1 | Abcam | | Cat# ab264350 |
| HRP-conjugated Goat anti-Mouse IgG | Proteintech | | Cat# SA00001-1 |
| HRP-conjugated Goat anti-Rabbit IgG | Bioworld | | Cat# SA00001-2 |
| Chemicals, Peptides, and Recombinant Proteins | | | |
| Recombinant murine GM-CSF | Peprotech | | Cat# 315-03 |
| Recombinant murine IL-4 | Peprotech | | Cat# 214-14 |
| Recombinant murine IL-2 | Peprotech | | Cat# 212-12 |
| TLR2 antagonist (TLR2-IN-C29) | Selleck | | Cat# S6597 |
| TLR4 antagonist (MD2-IN-1) | Selleck | | Cat# S6573 |
| Fetal bovine serum | Sigma | | Cat# F2442 |
| RPMI 1640 Medium | GIBCO | | Cat# 21875034 |
| Collagenase | Sigma | | Cat# C9263 |
| LPS (derived from *E. coli*) | Sigma | | Cat# L2630 |
| DNase I | Roch | | Cat# 10104159001 |
| TruStain FcX | Biolegend | | Cat# 101320 |
| Cell Stimulation Cocktail | eBioscience | | Cat# 00-4975-93 |
| Lipofectamine 3000 | Invitrogen | | Cat# L3000015 |
| Ampicillin | Aladdin | | Cat# A433389 |
| Neomycin | Aladdin | | Cat# N109017 |
| Vancomycin | Aladdin | | Cat# V301569 |
| Metronidazole | Aladdin | | Cat# M432726 |
| AOM | MP Biomedicals | | Cat# 25843-45-2 |
| DSS | MP Biomedicals | | Cat# 9011-18-1 |
| FITC-dextran | Sigma | | Cat# FD4 |
| Critical Commercial Assays | | | |
| CD4+ T cell Isolation Kit | Miltenyi Biotec | | Cat# 130-104-454 |
| Mouse LPS ELISA Kit | Coibo Bio | | Cat# CB10838-Mu |
| QIAamp Fast DNA Stool Mini Kit | Qiagen | | Cat# 51604 |
| cDNA Synthesis Kit | Yeasen | | Cat# 11149ES60 |
| qPCR SYBR Green Master Mix | Yeasen | | Cat# 11201ES08 |
| Fixation/Permeabilization Kit | BD Biosciences | | Cat# 554714 |
| Alcian Blue & Nuclear Fast Red Staining Kit | Beyotime | | Cat# C0153S |
| ChIP Kit | Bioruqi | | Cat# RQM006 |
| Bacterial LPS Extraction Kit  Biotin Quick Labeling Kit with Biotin-LC-NHS  Biotinylated Protein Pull-down Kit | Solarbio  Beyotime  Beyotime | | Cat# EX1740  Cat# P0632  Cat# P0654 |
| UltraSensitiveTM SP IHC Kit | Maxim | | Cat# KIT-9720 |
| Deposited Data |  | |  |
| Fecal metagenomic sequencing | GSA | | HRA005038 |
| RNA-sequencing | GEO | | GSE279592 |
| Experimental Models: Animals, Strains | |  |  |
| *Tlr4*^-/-^ mice | | Gempharmatech | N/A |
| *Megasphaera elsdenii* | | CCUG | Cat# 64197T |
| Oligonucleotides | | | |
| Primers for qRT-PCR | | | |
| Primer: Irf4 (FWD): GGAAGACAAGATTACGATGTGC (5'-3') | This paper | | N/A |
| Primer: Irf4 (REV): AATCCTGTACACCTTGTATGGG (5'-3') | This paper | | N/A |
| Primer: Tbx21 (FWD): ATCACTAAGCAAGGACGGCGAATG (5'-3') | This paper | | N/A |
| Primer: Tbx21 (REV): ACCAAGACCACATCCACAAACATCC (5'-3') | This paper | | N/A |
| Primer: Gata3 (FWD): ATTACCACCTATCCGCCCTAT (5'-3') | This paper | | N/A |
| Primer: Gata3 (REV): CGGTTCTGCCCATTCATTTTAT (5'-3') | This paper | | N/A |
| Primer: Rorc (FWD): ACAAATTGAAGTGATCCCTTGC (5'-3') | This paper | | N/A |
| Primer: Rorc (REV): GGAGTAGGCCACATTACACTG (5'-3') | This paper | | N/A |
| Primer: Foxp3 (FWD): TTTCACCTATGCCACCCTTATC (5'-3') | This paper | | N/A |
| Primer: Foxp3 (REV): CATGCGAGTAAACCAATGGTAG (5'-3') | This paper | | N/A |
| Primer: Ifng (FWD): CTGGAGGAACTGGCAAAAGGATGG (5'-3') | This paper | | N/A |
| Primer: Ifng (REV): GACGCTTATGTTGTTGCTGATGGC (5'-3') | This paper | | N/A |
| Primer: Il4 (FWD): TACCAGGAGCCATATCCACGGATG (5'-3') | This paper | | N/A |
| Primer: Il4 (REV): TGTGGTGTTCTTCGTTGCTGTGAG (5'-3') | This paper | | N/A |
| Primer: Il17a (FWD): GAGCTTCATCTGTGTCTCTGAT (5'-3') | This paper | | N/A |
| Primer: Il17a (REV): GCCAAGGGAGTTAAAGACTTTG (5'-3') | This paper | | N/A |
| Primer: Il10 (FWD): TTCTTTCAAACAAAGGACCAGC (5'-3') | This paper | | N/A |
| Primer: Il10 (REV): GCAACCCAAGTAACCCTTAAAG (5'-3') | This paper | | N/A |
| Primer: Il1b (FWD): CACTACAGGCTCCGAGATGAACAAC (5'-3') | This paper | | N/A |
| Primer: Il1b (REV): TGTCGTTGCTTGGTTCTCCTTGTAC (5'-3') | This paper | | N/A |
| Primer: Il23 (FWD): CAGCGGGACATATGAATCTACT (5'-3') | This paper | | N/A |
| Primer: Il23 (REV): TTGAAGATGTCAGAGTCAAGCA (5'-3') | This paper | | N/A |
| Primer: Tnf (FWD): CACCACGCTCTTCTGTCTACTGAAC (5'-3') | This paper | | N/A |
| Primer: Tnf (REV): TGGGCTACGGGCTTGTCACTC (5'-3') | This paper | | N/A |
| Primer: Megasphaera elsdenii (FWD): AGATGGGGACAACAGCTGGA (5'-3') | This paper | | N/A |
| Primer: Megasphaera elsdenii (REV): CGAAAGCTCCGAAGAGCCT (5'-3') | This paper | | N/A |
| Primer: 16s rRNA (FWD): TCCTACGGGAGGCAGCAGT (5'-3') | This paper | | N/A |
| Primer: 16s rRNA (REV): GGACTACCAGGGTATCTAATCCTGTT (5'-3') | This paper | | N/A |
| Primers for ChIP-qPCR |  | |  |
| Primer: Il1b (FWD): AGAGATGACAAACCAGGGAGG (5'-3') | This paper | | N/A |
| Primer: Il1b (REV): AGACCTGATGTACCTTTTTCCCC (5'-3') | This paper | | N/A |
| Primer: Il23 (FWD): AGGGATTCAGGTCAGGGAGT (5'-3') | This paper | | N/A |
| Primer: Il23 (REV): TGTGAGTTGGGTTCCTGTGT (5'-3') | This paper | | N/A |
| Primer: Cd80 (FWD): AGCCACGCTCTGGATAACCT (5'-3') | This paper | | N/A |
| Primer: Cd80 (REV): GTATGCTCCTAGCCGTCACAG (5'-3') | This paper | | N/A |
| Primer: Cd86 (FWD): AGTTAGGAGGCCCAAGTCCA (5'-3') | This paper | | N/A |
| Primer: Cd86 (REV): TCTTGACCCATCCCCACAGA (5'-3') | This paper | | N/A |

**Table S2.** **Score for DAI of colitis**

| Scoring Item | Scoring Grade ^a)^ | Description |
| --- | --- | --- |
| Body weight loss (%) | 0 | Normal |
|  | 1 | 1-5 |
|  | 2 | 5-10 |
|  | 3 | 10-20 |
|  | 4 | >20 |
| Occult/gross bleeding | 0 | Negative |
|  | 1 | - |
|  | 2 | Hemoccult + |
|  | 3 | - |
|  | 4 | Gross perianal bleeding |
| Stool consistency | 0 | Normal |
|  | 1 | - |
|  | 2 | Loose stool |
|  | 3 | - |
|  | 4 | Diarrhea |

^a)^ The DAI score was determined as the average of three item score.

**Table S3.** **Score for colonic** **inflammatory histopathology**

| Scoring Item | Scoring Grade ^a)^ | Description |
| --- | --- | --- |
| Submucosal edema | 0 | No pathological changes |
|  | 1 | Mild edema (the submucosa is <0.20 mm wide and accounts for <50% of the diameter of the entire intestinal wall |
|  | 2 | Moderate edema (the submucosa is 0.21 to 0.45 mm wide and accounts for 50 to 80% of the diameter of the entire intestinal wall) |
|  | 3 | Profound edema (the submucosa is >0.46 mm wide and accounts for >80% of the diameter of the entire intestinal wall) |
| Polymorphonuclear granulocytes (PMN) infiltration into the lamina propria | 0 | <5 PMN/high-power field |
|  | 1 | 5 to 20 PMN/high-power field |
|  | 2 | 21 to 60/high-power field |
|  | 3 | 61 to 100/high-power field |
|  | 4 | >100/high-power field |
| Goblet cells | 0 | >28 goblet cells/high-power field |
|  | 1 | 11 to 28 goblet cells/high-power field |
|  | 2 | 1 to 10 goblet cells/high-power field |
|  | 3 | <1 goblet cell/high-power field |
| Epithelial integrity | 0 | No pathological changes detectable |
|  | 1 | Epithelial desquamation |
|  | 2 | Erosion of the epithelial surface |
|  | 3 | Epithelial ulceration |

^a)^ The pathological score was determined as the sum of each item score, which ranges between 0 and 13 and levels of inflammation are assessed as follows: 0 = intestine intact without any signs of inflammation; 1 to 2 = minimal signs of inflammation; 3 to 4 = slight inflammation; 5 to 8 = moderate inflammation; and 9 to 13 = profound inflammation.

| **Table S4. The clinical characteristics of recruited subjects** | | | |  |
| --- | --- | --- | --- | --- |
|  | HC (n=222) | CRC_EO (n=125) | CRC_LO (n=151) | p-value |
| Gender, n (%) |  |  |  |  |
| Male | 98 (44) | 61 (49) | 95 (63) | 0.0205 |
| Female | 124 (56) | 64 (51) | 56 (37) |  |
| Age, median (95%CI) | 50 (48, 54) | 43 (41, 45) | 62 (60, 65) | <0.001 |
| Stage, n(%) |  |  |  |  |
| 0&I |  | 26 (21) | 41 (27) | 0.0103 |
| II |  | 20 (16) | 47 (31) |  |
| III |  | 49 (39) | 46 (31) |  |
| IV |  | 30 (24) | 17 (11) |  |
| Tumor location, n (%) |  |  |  |  |
| Right-hemicolon |  | 23 (18) | 35 (23) | 0.6391 |
| Left-hemicolon |  | 39 (31) | 40 (27) |  |
| Rectum |  | 63 (51) | 76 (50) |  |
| Differentiation, n (%) |  |  |  |  |
| Low |  | 35 (28) | 43 (28) | >0.9999 |
| Median & high |  | 90 (72) | 108 (72) |  |

| **Table S5. Differential taxa among healthy and CRC subjects resulted from fecal metagenomic sequencing (selected by P<0.01 and log2fc >1&<-1)** | | | | | | |
| --- | --- | --- | --- | --- | --- | --- |
| **Bacteria_Name** | **EO_vs_CTR_log2fc** | **EO_vs_CTR_pvalue** | **LO_vs_CTR_log2fc** | **LO_vs_CTR_pvalue** | **EO_vs_LO_log2fc** | **EO_vs_LO_pvalue** |
| Parvimonas | 2.339234365 | 0.073717397 | 2.676926158 | 0.009216567 | -0.337691793 | 0.668547542 |
| Porphyromonas | 1.726540151 | 0.047627833 | 2.619769987 | 0.008880173 | -0.893229836 | 0.265653258 |
| Fusobacterium | 1.577407297 | 0.001974297 | 1.943456992 | 0.049768805 | -0.366049695 | 0.571366427 |
| Escherichia | -0.659147994 | 0.065603593 | 0.58069966 | 0.089716653 | -1.239847653 | 0.001138748 |
| Collinsella | -1.120824788 | 0.001181289 | 0.028022213 | 0.959698087 | -1.148847001 | 0.1225053 |
| Natronobacterium | 1.103253962 | 0.002009164 | -0.049709934 | 0.895974623 | 1.152963896 | 0.001618485 |
| Salinarchaeum | 0.864066298 | 0.008646958 | -0.141426103 | 0.677985637 | 1.005492401 | 0.0040161 |
| Gryllotalpicola | 0.688216463 | 0.000599351 | -0.346378076 | 0.093824061 | 1.034594539 | 6.17473E-06 |
| Bifidobacterium | -1.34634035 | 0.000814979 | -0.359414634 | 0.354577028 | -0.986925716 | 0.023824561 |
| Weissella | -1.35734719 | 0.005063605 | -0.374203057 | 0.494035935 | -0.983144133 | 0.139606022 |
| Candidatus Cloacimonas | 0.923117876 | 0.037955 | -0.41445048 | 0.434737349 | 1.337568356 | 0.009519831 |
| Kytococcus | 0.605754302 | 0.016262132 | -0.43500009 | 0.067068041 | 1.040754392 | 0.000195345 |
| Tamlana | 0.578833003 | 0.015399321 | -0.452256489 | 0.018453239 | 1.031089492 | 0.000164336 |
| Sideroxydans | 0.535364974 | 0.003442482 | -0.476410351 | 0.020188186 | 1.011775325 | 3.19765E-06 |
| Lacunisphaera | 0.52319026 | 0.009057234 | -0.499002975 | 0.006517275 | 1.022193235 | 1.89816E-05 |
| Marmoricola | 0.518291461 | 0.002516018 | -0.506829747 | 0.001993818 | 1.025121208 | 2.95511E-07 |
| Rhizorhabdus | 0.537831647 | 0.008156772 | -0.536049426 | 0.015440939 | 1.073881073 | 5.80809E-05 |
| Ramlibacter | 0.45126271 | 0.126999016 | -0.555881753 | 0.000276576 | 1.007144463 | 0.004460988 |
| Halodesulfurarchaeum | 0.449028799 | 0.109657746 | -0.556463602 | 0.045481845 | 1.005492401 | 0.001452757 |
| Chania | 0.484075746 | 0.016824234 | -0.569519755 | 0.003764007 | 1.053595501 | 7.13989E-06 |
| Pleomorphomonas | 0.478141276 | 0.017445548 | -0.569832806 | 0.000460029 | 1.047974082 | 2.25782E-05 |
| Methylosinus | 0.555404634 | 0.004103501 | -0.576800701 | 0.001222687 | 1.132205335 | 1.42215E-06 |
| Selenomonas | 0.798852738 | 0.080432963 | -0.579181759 | 0.000914055 | 1.378034497 | 0.012083588 |
| Leptolyngbya | 0.411577835 | 0.155451013 | -0.60314379 | 0.032287431 | 1.014721626 | 0.001928472 |
| Halobellus | 0.413954801 | 0.026533403 | -0.603467087 | 0.001754683 | 1.017421888 | 9.11803E-06 |
| Beutenbergia | 0.376272456 | 0.0529097 | -0.628413444 | 0.00124647 | 1.0046859 | 4.24655E-05 |
| Nitrospirillum | 0.472179536 | 0.00543628 | -0.632545063 | 4.59644E-05 | 1.104724599 | 1.0788E-07 |
| Acidihalobacter | 0.370104302 | 0.048828693 | -0.634530045 | 0.000139143 | 1.004634347 | 8.89802E-06 |
| Microcystis | 0.389558147 | 0.008581361 | -0.647735542 | 2.30745E-05 | 1.037293688 | 1.17753E-07 |
| Cellulosimicrobium | 0.375914512 | 0.031638092 | -0.65603935 | 0.0001314 | 1.031953862 | 1.16048E-06 |
| Congregibacter | 0.589568875 | 0.017183633 | -0.658113678 | 0.009613957 | 1.247682553 | 0.000129536 |
| Paraphotobacterium | 0.354792729 | 0.209972957 | -0.666381294 | 0.016030322 | 1.021174023 | 0.004231678 |
| Kordia | 0.390205918 | 0.203545217 | -0.667821829 | 0.021027744 | 1.058027747 | 0.003201149 |
| Iodobacter | 0.451654268 | 0.255766141 | -0.675314419 | 0.000791919 | 1.126968687 | 0.022062488 |
| Chthonomonas | 0.404483064 | 0.191972569 | -0.684996972 | 0.059455021 | 1.089480036 | 0.003576019 |
| Egicoccus | 0.359890596 | 0.022542866 | -0.689258999 | 4.79588E-05 | 1.049149596 | 1.16507E-07 |
| Paeniclostridium | -1.183411673 | 0.007462948 | -0.746848025 | 0.155862762 | -0.436563648 | 0.454384706 |
| Euzebyella | 0.364120531 | 0.21823792 | -0.765916968 | 0.011938445 | 1.130037499 | 0.000984388 |
| Curvibacter | 0.451177265 | 0.060172589 | -0.765916968 | 2.24044E-06 | 1.217094233 | 6.25263E-05 |
| Egibacter | 0.249802625 | 0.184714117 | -0.765916968 | 2.37673E-05 | 1.015719593 | 6.38851E-06 |
| Candidatus Ruthia | 0.584718823 | 0.080586258 | -0.773490188 | 0.0345775 | 1.35820901 | 0.002560663 |
| Natronomonas | 0.260026299 | 0.197503012 | -0.775503017 | 0.00032827 | 1.035529316 | 5.16632E-05 |
| Roseibacterium | 0.317184804 | 0.116118427 | -0.795752197 | 4.28563E-06 | 1.112937002 | 3.37618E-05 |
| Paraoerskovia | 0.454094749 | 0.020843684 | -0.807393604 | 7.08173E-05 | 1.261488352 | 6.9822E-07 |
| Chloroflexus | 0.218731179 | 0.368317689 | -0.81595765 | 0.001215733 | 1.03468883 | 0.00010762 |
| Marivivens | 0.329257637 | 0.318542663 | -0.817447269 | 0.003803182 | 1.146704905 | 0.003895763 |
| Tolumonas | 0.225137067 | 0.361078855 | -0.818531594 | 0.001059388 | 1.043668661 | 0.000703878 |
| Calditerrivibrio | 0.382651369 | 0.301241824 | -0.8190949 | 0.047712058 | 1.201746269 | 0.005923829 |
| Candidatus Nanopelagicus | 0.537372203 | 0.066828808 | -0.822500496 | 0.001136616 | 1.359872699 | 0.00015429 |
| Yoonia | 0.365199952 | 0.185799807 | -0.83957401 | 0.00149708 | 1.204773961 | 0.000253503 |
| Ferriphaselus | 0.601432696 | 0.016242973 | -0.867200304 | 0.001238111 | 1.468633 | 6.46685E-06 |
| Bacterioplanes | 0.218262848 | 0.417420265 | -0.873300503 | 0.001293683 | 1.091563351 | 0.000446446 |
| Nitratiruptor | 0.410354639 | 0.185069416 | -0.885826432 | 0.002009761 | 1.296181071 | 0.000965169 |
| Pyrococcus | 0.247877525 | 0.171534515 | -0.888773716 | 5.77634E-07 | 1.136651241 | 8.09909E-07 |
| Isosphaera | 0.197376139 | 0.499066447 | -0.919722304 | 0.002501326 | 1.117098443 | 0.000902324 |
| Wolbachia | 0.25324633 | 0.359551695 | -0.920601651 | 0.00136118 | 1.173847981 | 0.000325607 |
| Kerstersia | 0.634961481 | 0.156481071 | -0.931485564 | 0.003955906 | 1.566447046 | 0.004709953 |
| Acaryochloris | 0.227776319 | 0.537544788 | -0.949516906 | 0.02617008 | 1.177293225 | 0.001219996 |
| Megasphaera | 3.05968551 | 0.002747446 | -0.995102681 | 0.00064159 | 4.054788191 | 0.001403749 |
| Sulfurovum | -0.077318284 | 0.657773283 | -1.028951374 | 5.13449E-06 | 0.95163309 | 0.000530824 |
| Dichelobacter | -0.002239167 | 0.989711287 | -1.028951374 | 3.57174E-05 | 1.026712207 | 4.94632E-05 |
| Ruminococcus | 0.199023883 | 0.612357784 | -1.036564397 | 0.001592645 | 1.23558828 | 0.015621866 |
| Histophilus | 0.014504097 | 0.983279587 | -1.048733986 | 0.000841262 | 1.063238083 | 0.025395736 |
| Metakosakonia | -0.120786312 | 0.626651961 | -1.048850931 | 0.0000355 | 0.928064619 | 0.001174301 |
| Clostridium | -0.724189944 | 0.003529534 | -1.051405865 | 0.000167869 | 0.327215921 | 0.033092776 |
| Acetoanaerobium | -2.24261012 | 7.89756E-05 | -1.068212035 | 0.017183973 | -1.174398085 | 0.111373783 |
| Jonesia | 0.090197809 | 0.842225039 | -1.068479738 | 0.018413268 | 1.158677547 | 0.007383314 |
| Candidatus Saccharimonas | -0.474982676 | 0.029051202 | -1.097993018 | 3.33609E-06 | 0.623010342 | 0.051137963 |
| Intrasporangium | 0.07940722 | 0.750774548 | -1.101707716 | 5.44841E-07 | 1.181114936 | 5.14595E-05 |
| Anaplasma | -0.196492222 | 0.39448407 | -1.10333357 | 0.000433345 | 0.906841348 | 0.006856427 |
| Thermanaeromonas | -0.12661358 | 0.525130239 | -1.115366126 | 5.01779E-08 | 0.988752545 | 1.44412E-05 |
| Actinotignum | -0.486209931 | 0.502601701 | -1.12656217 | 0.212396109 | 0.640352239 | 0.00402131 |
| Desulfocapsa | 0.061391939 | 0.811501975 | -1.130622792 | 9.97755E-06 | 1.192014731 | 1.84334E-06 |
| Gloeocapsa | 0.350414288 | 0.258752612 | -1.166454897 | 0.000087624 | 1.516869186 | 0.000250375 |
| Crinalium | -0.33224764 | 0.266517478 | -1.167471238 | 5.26087E-06 | 0.835223598 | 0.069968472 |
| Auricoccus | -0.586045199 | 0.03085997 | -1.16981391 | 1.87623E-06 | 0.583768711 | 0.131374464 |
| Devriesea | -0.251702933 | 0.264234491 | -1.198876375 | 5.27004E-05 | 0.947173442 | 0.001163101 |
| Kingella | 0.209453366 | 0.486266723 | -1.221596452 | 0.000282569 | 1.431049817 | 0.000072439 |
| Candidatus Thioglobus | -0.45478724 | 0.044232132 | -1.224422496 | 2.25682E-06 | 0.769635256 | 0.009748025 |
| Hydrogenobacter | 0.108415667 | 0.734933543 | -1.228260182 | 0.000378134 | 1.336675849 | 0.001258674 |
| Roseburia | -0.520213835 | 0.082940296 | -1.232914298 | 3.21544E-09 | 0.712700463 | 0.113211693 |
| Turicibacter | -0.66892151 | 0.073351081 | -1.271526343 | 0.000265984 | 0.602604833 | 0.20591024 |
| Salinigranum | -0.002811098 | 0.942527026 | -1.272222525 | 4.24489E-10 | 1.269411427 | 1.78391E-05 |
| Caproiciproducens | -1.216200636 | 4.96071E-05 | -1.426101122 | 0.000024363 | 0.209900486 | 0.630262214 |
| Faecalibacterium | -1.287678952 | 1.44575E-16 | -1.478262524 | 5.13083E-18 | 0.190583572 | 0.360524528 |
| Desulfurella | -0.420544676 | 0.237658242 | -1.525782964 | 0.000016995 | 1.105238288 | 0.040137012 |
| Ethanoligenens | -1.642062681 | 0.000582111 | -1.586688015 | 0.001038035 | -0.055374666 | 0.909141675 |
| Chondrocystis | -0.1494385 | 0.706402677 | -1.65603935 | 0.000231597 | 1.50660085 | 0.018510953 |
| Solibacillus | -0.965000254 | 0.186989191 | -1.689354631 | 0.060046641 | 0.724354377 | 0.002152627 |
| Megamonas | -0.040965824 | 0.933057278 | -1.760616995 | 0.009288396 | 1.719651171 | 0.071481524 |
| Dialister | -1.102114734 | 0.013814149 | -2.083763344 | 0.000133423 | 0.98164861 | 0.100188488 |
| Geosporobacter | -1.355612039 | 0.034192038 | -2.642214983 | 0.001054322 | 1.286602944 | 0.123774826 |
| Fibrobacter | -2.710351815 | 0.323132133 | -3.489225278 | 0.288551543 | 0.778873463 | 2.02759E-05 |
| Acidimicrobium | -3.943118485 | 0.331637832 | -4.805500249 | 0.317023436 | 0.862381764 | 0.008906854 |
